# Deciphering the molecular and cellular atlas of immune cells in septic patients with different bacterial infections

**DOI:** 10.1186/s12967-023-04631-4

**Published:** 2023-11-02

**Authors:** Ping Sun, Mintian Cui, Jiongjie Jing, Fanyu Kong, Shixi Wang, Lunxian Tang, Junling Leng, Kun Chen

**Affiliations:** 1 Translational Medical Center for Stem Cell Therapy, Institute for Regenerative Medicine, School of Life Sciences and Technology, Shanghai East Hospital, Tongji University, Shanghai, 200127 China; 2 Department of Emergency, Affiliated Hospital of Yangzhou University, Yangzhou, 225000 China; 3 Department of Internal Emergency Medicine, Shanghai East Hospital, Tongji University School of Medicine, Shanghai, 200120 China; 4 Shanghai Key Laboratory of Signaling and Disease Research, Frontier Science Center for Stem Cell Research, School of Life Sciences and Technology, Tongji University, Shanghai, 200092 China

**Keywords:** Sepsis, Various bacterial infections, Multi-omics, Cellular heterogeneity, Stratified targeted therapies

## Abstract

**Background:**

Sepsis is a life-threatening organ dysfunction caused by abnormal immune responses to various, predominantly bacterial, infections. Different bacterial infections lead to substantial variation in disease manifestation and therapeutic strategies. However, the underlying cellular heterogeneity and mechanisms involved remain poorly understood.

**Methods:**

Multiple bulk transcriptome datasets from septic patients with 12 types of bacterial infections were integrated to identify signature genes for each infection. Signature genes were mapped onto an integrated large single-cell RNA (scRNA) dataset from septic patients, to identify subsets of cells associated with different sepsis types, and multiple omics datasets were combined to reveal the underlying molecular mechanisms. In addition, an scRNA dataset and spatial transcriptome data were used to identify signaling pathways in sepsis-related cells. Finally, molecular screening, optimization, and de novo design were conducted to identify potential targeted drugs and compounds.

**Results:**

We elucidated the cellular heterogeneity among septic patients with different bacterial infections. In *Escherichia coli* (*E. coli*) sepsis, 19 signature genes involved in epigenetic regulation and metabolism were identified, of which *DRAM1* was demonstrated to promote autophagy and glycolysis in response to *E. coli* infection. *DRAM1* upregulation was confirmed in an independent sepsis cohort. Further, we showed that *DRAM1* could maintain survival of a pro-inflammatory monocyte subset, C10_ULK1, which induces systemic inflammation by interacting with other cell subsets via resistin and integrin signaling pathways in blood and kidney tissue, respectively. Finally, retapamulin was identified and optimized as a potential drug for treatment of *E. coli* sepsis targeting the signature gene, *DRAM1*, and inhibiting *E. coli* protein synthesis. Several other targeted drugs were also identified in other types of sepsis, including nystatin targeting *C1QA* in *Neisseria* sepsis and dalfopristin targeting *CTSD* in *Streptococcus viridans* sepsis.

**Conclusion:**

Our study provides a comprehensive overview of the cellular heterogeneity and underlying mechanisms in septic patients with various bacterial infections, providing insights to inform development of stratified targeted therapies for sepsis.

**Supplementary Information:**

The online version contains supplementary material available at 10.1186/s12967-023-04631-4.

## Introduction

Sepsis is a life-threatening organ dysfunction primarily caused by dysregulated host immune responses against infection [1, 2]. Notably, bacteria are the primary infection source during sepsis [3]. Clinically, symptoms and phenotypes differ among septic patients with different types of bacteria [4]. However, knowledge of septic patients with different bacterial infections remains limited, impeding precise treatment.

Significant transcriptional differences have been reported in sepsis caused by different bacterial infections [5, 6]. Genes highly expressed in sepsis caused by *Burkholderia pseudomallei* are related to inflammatory responses, apoptosis, and cellular metabolic processes [5]. *Escherichia coli*-induced sepsis is associated with fructose and mannose metabolism [6]. However, there has been limited systematic investigation into the transcriptional differences among distinct types of bacteria-induced sepsis. Critically, bulk transcriptomic analysis is insufficient to fully elucidate the cellular heterogeneity of sepsis with different bacterial infections.

The emergence of single-cell transcriptomics provides an opportunity to study sepsis at the cellular level [7]. Recently, single-cell studies on sepsis have discovered disease-associated cytologic signatures of bacterial sepsis [8] and tracked temporal expression changes in specific cell types in patients surviving from or with fatal sepsis [9]. However, studies that rely solely on single-cell RNA (scRNA) sequencing data to investigate the characteristics of septic patients with different infections have been limited by small sample size. Therefore, integrating bulk and scRNA data to study different types of bacterial sepsis at the cellular level has potential to be a fruitful approach [10]. Using large-scale bulk RNA-seq data to identify global signature genes and then investigating cellular heterogeneity using scRNA data, will provide a new perspective and comprehensive understanding of different types of bacterial sepsis. Moreover, integration of these two types of transcriptomic data allows for mutual validation, increasing the accuracy of results.

In addition to immune cell heterogeneity, the spatial localization of immune cells is another key factor determining differences in response to bacterial infections, as cell functions are influenced by neighboring cells and signals in the tissue microenvironment [11]. In the fields of tumor and developmental biology, spatial transcriptomics (ST) has been widely used to understand cellular interactions in microenvironments and decipher location-associated mechanisms of tissue formation and organogenesis [12, 13]. Recent research based on kidney tissue scRNA and ST data from a mouse model of sepsis revealed that dysregulated cell–cell communication is a major contributor to sepsis [14]. Additionally, subcellular spatial sequencing can provide information on the locations of gene expression within a cell [15], which can improve understanding of the molecular mechanisms underlying different bacterial sepsis subtypes.

In this study, we integrated multi-omics data, including bulk RNA, scRNA, spatial and subcellular ST, metabolomics, and cheminformatics datasets, to identify specific signature gene sets, cell clusters, molecular mechanisms, and underlying targeted drugs in septic patients with different bacterial infections. Our findings provide an overall perspective on the heterogeneity of sepsis with different bacterial infections and will inform the development of personalized treatment and management strategies.

## Materials and methods

### Datasets

Three sepsis blood transcription profile datasets (GSE69528, GSE13015, and GSE4607) annotated with detailed pathogen information were retrieved from Gene Expression Omnibus (GEO) at the National Center for Biotechnology Information. Signature genes of patients with different types of sepsis were identified based on these datasets. Gene expression profiles from whole blood cells infected by bacteria ex vivo (GSE65088) were used to identify the basic features of *E. coli* infection. The role of *BCL6* in cell differentiation was investigated using gene expression data from myeloid cells from wild type (WT) and *BCL6*-knockout (KO) mice (GSE24813). Three scRNA-seq datasets (GSE151263, GSE167363, and SCP548) from published studies of septic patients were downloaded, integrated, and the integrated dataset used to identify specific cells related to septic patients and different bacterial infections. ST data from healthy human (GSE171406) and mouse sepsis model (GSE154107) kidney tissue sections were used to investigate the functions and signaling pathways of relevant cell subpopulations within kidney. ST data from mice were integrated with scRNA data (GSE151658) generated by the same study. Furthermore, the nCounter Elements TagSets sequencing-based dataset (GSE167914) was used to examine changes in enzymes related to mitochondrial respiration and function in the peripheral blood of septic patients and healthy controls. Metabolomics data from septic patients (MTBLS563) were also used to validate metabolic changes identified in these patients. Subcellular co-localization analysis was conducted based on a sequential fluorescence in situ hybridization-plus (seqFISH-plus) dataset (https://github.com/CaiGroup/seqFISH-PLUS), to reveal the functional relationships between co-expressed genes. The regulatory role of *ELF1* in *DRAM1* expression was verified using chromatin immunoprecipitation followed by sequencing (ChIP-seq) data (GSE122203). Details of all datasets used in this study are summarized in Table 1.

**Table 1:** Details of the datasets used in this study

Dataset	Type	Platform	Septic patients/Case	Control	References
GSE69528	Bulk RNA	GPL10558	83	55	Pankla R et al. [5]
GSE13015	Bulk RNA	GPL6947	29	10	
		GPL6106	48	19	
GSE4607	Bulk RNA	GPL570	108	15	Wong HR et al. [16]
GSE65088	Bulk RNA	GPL10558	36	21	Dix A et al. [17]
GSE24813	Bulk RNA	GPL1261	4	6	Hurtz C et al. [18]
GSE151263	scRNA	GPL20301	7	0	Jiang Y et al. [19]
GSE167363	scRNA	GPL24676	10	2	Qiu X et al. [9]
SCP548	scRNA	GPL29783	36	29	Reyes M et al. [8]
GSE171406	ST	GPL24676	2	2	Melo Ferreira R et al. [20]
GSE154107	ST	GPL24247	1	0	Janosevic D et al. [14]
GSE151658	scRNA	GPL24247	6	1	Janosevic D et al. [14]
GSE167914	RNA	GPL29783	28	11	Herwanto V et al
MTBLS563	NMR-based metabolic profiling	/	55	58	Grauslys A et al. [21]
seqFISH-PLUS	seqFISH-plus	/	/	/	Eng. et al. 2019. [22]
GSE122203	ChIP-seq	GPL16791	2	2	Seifert LL et al

*NMR* Nuclear magnetic resonance

### Human samples

The study was approved by the research ethics board of Shanghai East Hospital (ChiCTR2000035722). Informed consent was obtained from all participants in this study. Subjects with sepsis were diagnosed using the sepsis 3.0 criteria [23]. Peripheral blood mononuclear cells (PBMCs) were collected for gene expression analysis. Patients exhibiting life-threatening organ dysfunction, indicated by an increase of at least two points in the Sequential Organ Failure Assessment (SOFA) score following infection, were included. Patients with conditions including HIV infection, autoimmune diseases, hematological malignancies, and viral hepatitis were excluded. The case group and the control group were matched for age and sex. Information on the case–control subjects enrolled in this study is provided in Table 2.

**Table 2:** Demographic and clinical characteristics of septic patients and healthy controls

Characteristics	Healthy control (*n* = 4)	Sepsis (*n* = 5)
Demographic characteristics		
Female/male	2/2	3/2
Median age (years) (IQR)	72 (67–76)	75.6 (59–86)
Site of infection		
Lung	/	2/5
Abdominal	/	1/5
Blood	/	/
Others	/	2/5
SOFA score, median (IQR)		5.8 ± 1.4
Laboratory Tests		/
CRP (mg/L), mean ± SEM	/	152.83 ± 19.69
WBC (10^9^/L), mean ± SEM	/	1.22 ± 11.63
PCT (ng/mL), median (IQR)	/	5.26 ± 10.06
PLT (10^9^/L), mean ± SEM	/	178.4 ± 35.83
Lactate (mg/L), mean ± SEM	/	0.29 ± 1.48
Immunologic parameter	/	
Lymphocytes, × 10^9^/L	/	0.61 ± 0.09
Monocytes, × 10^9^/L	/	0.69 ± 0.13
Neutrophil, × 10^9^/L	/	10.28 ± 1.13
IgG, mg/dl	/	1009.8 ± 82.40
IgM, mg/dl	/	98.8 ± 9.93
Complications, N (%)		
Acute respiratory failure	/	2 (40)
Acute cardiac dysfunction	/	3 (60)
Acute kidney injury	/	3 (60)
Acute hepatic insufficiency	/	1 (20)
28-day mortality, N (%)	/	2 (40)
Hospital mortality, N (%)	/	2 (40)

*SOFA* Sequential Organ Failure Assessment, *CRP* C-Reactive Protein, *PCT* Procalcitonin, *WBC* White Blood Cell count, *PLT* Platelet count

### Quantitative real-time PCR

Total RNA was isolated from PBMCs using the RNAfast200 kit (Fastagen, China, #220011), according to the manufacturer’s instructions. cDNA was synthesized from total RNA using Evo M-MLV RT Premix for qPCR (ACCURATE BIOLOGY, China, #AG11706), according to the manufacturer’s instructions. Quantitative real-time PCR (qRT-PCR) was conducted using SYBR Green Premix Pro Taq HS qPCR Kits (ACCURATE BIOLOGY, China, #AG11701). To ensure the accuracy of qRT-PCR results, all RNA samples were extracted under consistent conditions, and equal amounts of RNA were used for reverse transcription to cDNA. Cycle threshold (CT) values of target genes were determined, and relative expression calculated using the 2^−ΔCT^ method. Data were normalized using the reference gene encoding β-actin, to correct for potential sample-to-sample variation. Primers used for qRT-PCR are listed in Table 3.

**Table 3:** Primers used for qPCR in this study

Gene		Primer sequence (5ʹ → 3ʹ)
Human *DRAM1*	Forward	ATT GGT GGG ATG TTT CGG AAT GG
Human *DRAM1*	Reverse	TGA TGG ACT GTA GGA GCG TGT AC
Human *LDHA*	Forward	GTG TGC CTG TAT GGA GTG GA
Human *LDHA*	Reverse	GPC IAA CCA CCT GCT TGT GAA CCT
Human *ACTB*	Forward	CAT GTA CGT TGC TAT CCA GGC
Human *ACTB*	Forward	CTC CTT AAT GTC ACG CAC GAT

### Identification of signature gene sets for different types of sepsis

Signature gene sets for different types of sepsis were identified using the following criteria. First, differentially expressed genes (DEGs) between septic patients with different bacterial infections and healthy control samples in each dataset were identified using the limma package [24], with *P* correction using the Benjamini–Hochberg method. To ensure the reliability of the results, only sepsis types with more than three samples in each dataset were compared. For each type of sepsis, differential results of multiple datasets were corrected using the robust rank aggregation (RRA) method for those included in more than one dataset, to obtain a comprehensive ranking [25]. Finally selected genes that were significantly differentially expressed (|Log_2_ *FC*|> 1 and adjusted *P* < 0.05) in each dataset, as well as significant after RRA correction, were identified as potential key genes for each type of sepsis (*P* < 0.05). If only one dataset contained patients with this type of sepsis, the threshold was increased (|Log_2_ *FC*|> 2 and adjusted *P* < 0.05), to improve the reliability of the result. Of selected potential key genes, those that were upregulated were considered signature genes. Signature genes were also validated using independent datasets and receiver operating characteristic curves plotted.

### Functional enrichment analysis

Gene Ontology (GO) and pathway enrichment analyses were performed using Metascape (https://metascape.org) and Database for Annotation, Visualization and Integrated Discovery (DAVID) (https://david.ncifcrf.gov/).

### Deconvolution analysis

Deconvolution analysis can be used to evaluate the proportions of different cell types in bulk data. Computational Methods for Immune Cell-Type Subsets (ComICS) (https://github.com/cran/ComICS), a computational method for cell-type subset analysis, was used for deconvolution analysis of septic patients. For deconvolution analysis of BCL6-KO data. CIBERSORTx was applied to generate a group feature matrix based on integrated single-cell data [26], and then estimated changes in the proportions of cell groups between WT and *BCL6*-KO data using this matrix.

### Single cell RNA-seq data analysis

Seurat was used to integrate the three scRNA datasets and Harmony was applied to correct for batch effects. Seurat is a widely used R package for scRNA-seq analysis [27] and Harmony is an excellent tool for integrating single cell data by mapping cells into a shared embedding [28]. Dimensionality reduction and clustering were then performed based on Harmony-corrected data.

Differential expression analysis between clusters and identification of putative marker genes for each cluster were used to annotate clusters. Given known cell markers, clusters were annotated based on expression of these putative marker genes. To determine the percentage of each cell type, the number of cells belonging to each type within a group was counted and divided by the total number of cells assigned to the group.

Signature enrichment analysis was performed using the irGSEA package (https://github.com/chuiqin/irGSEA/). To improve the robustness of the results, three methods (AUCell, UCell, and singcore) provided in irGSEA were used to calculate signature gene enrichment scores. After RRA correction, clusters in which scores were specifically upregulated in patients were selected as specific clusters related to different types.

The monocyte cell lineage trajectory was inferred using Monocle2, a semi-supervised analysis mode suitable for personalized analysis of cell clusters [29]. Pseudotemporal analysis was conducted to identify differentiation-related genes.

SCENIC (Single-Cell Regulatory Network Inference and Clustering) is a computational method used to infer gene regulatory networks in scRNA-seq data [30]. In this study, SCENIC was used to identify upstream transcription factors that regulate signature genes in relevant cell subpopulations.

CellChat was employed to investigate cell–cell interactions in both control and sepsis samples by detecting significant ligand-receptor pairs [31]. Communication between different cell types was identified by analyzing gene expression of ligands in one cell cluster and specific receptors in another. Cell communication scores were then calculated by taking the average expression levels of ligand-receptor genes.

### ST data analysis

For analysis of the human kidney tissue ST dataset, the FindTransferAnchors and TransferData functions of Seurat were applied to map the cell subtypes identified as associated with different sepsis types to the spatial data. Co-localization of cells in spatial positions was evaluated by analyzing neighboring cells of relevant cells and assessing the receptor-ligand interactions of cells in the tissue using SpaGene [32].

For analysis of single-cell and ST data from mouse kidney tissue, the two types of data were integrated using the Spatial Transcriptomics Deconvolution by Topic Modeling (STRIDE) method [33]. Using the integrated data, Multiview Intercellular SpaTial modeling framework (MISTy) was employed to analyze cell interactions. MISTy is a multi-view framework (intrinsic, local niche view, and tissue view) for modeling intercellular interactions from spatial data [34].

Based on seqFISH-plus data, Bento (https://github.com/ckmah/bento-tools) was used for subcellular spatial analysis and visualization. The distance between genes was defined as the average shortest distance.

### Metabolomics data analysis

Metabolic data from septic patients were downloaded from Metabolights (https://www.ebi.ac.uk/metabolights/) and analyzed using the online tool, MetaboAnalyst (5.0) [35]. Data were filtered and normalized, followed by one-factor statistical analysis. Additionally, fold-change and *P* values of metabolites between healthy controls and patients were calculated to identify significantly different metabolites.

### ChIP-seq data analysis

An *ELF1* ChIP-seq dataset was downloaded from GEO and ChIP-seq reads aligned to the hg19 genome assembly using bowtie2. The SAM file generated by bowtie2 was then converted to a BAM file using samtools. BAM files were subsequently transformed into BED files using bedtools. Peaks were identified using MACS, which operates based on the Poisson distribution, with a stringent q-value cut-off (0.0001). Results were visualized using Integrative Genomics Viewer (IGV) [36].

### Virtual screening and de novo design

Structural data of compounds and proteins were downloaded from ZINC20 [37] and UniProt, respectively. Batch docking was performed to identify compounds that may bind to the proteins using Vina [38]. Molecular optimization and de novo design were conducted using the online PanGu Drug database [39].

### Statistical methods

Test methods used are described in the figure legends. *P* < 0.05 was considered statistically significant (*P < 0.05, **P < 0.01, ***P < 0.001, ****P < 0.0001). Raw and adjusted *P* values are provided in the figures and tables. Analyses were mainly implemented in R language, based on version 4.0.2 (patched) (mainly for bulk RNA analyses) and 4.2.2 (mainly for single-cell analyses). In addition, seqFISH-plus and ST data were analyzed using Python language (version 3.9.12).

## Results

### Dysregulated genes in septic patients with different bacterial infections differ in functions

**Fig. 1:**
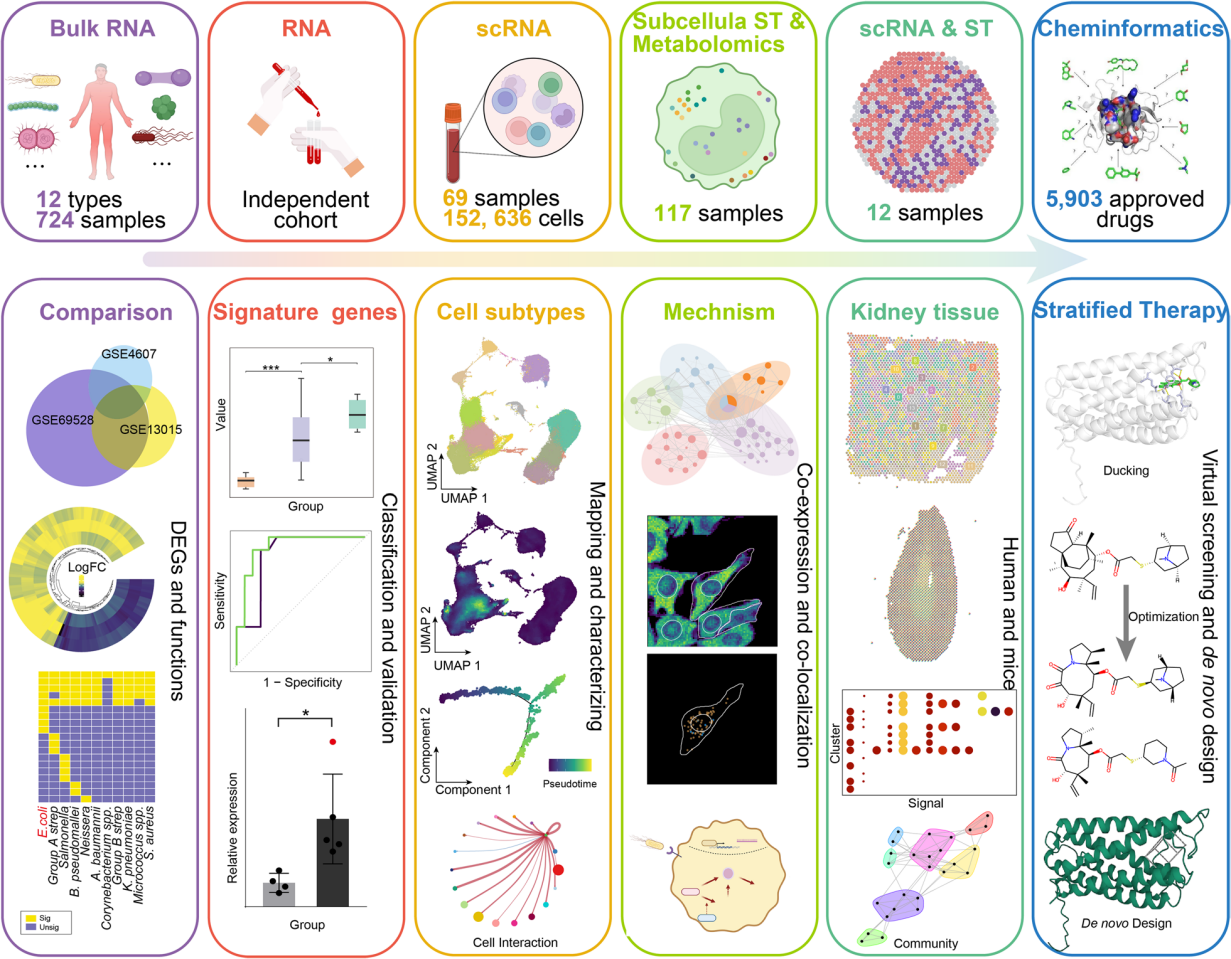
The experimental strategy is illustrated in a schematic map

**Fig. 2:**
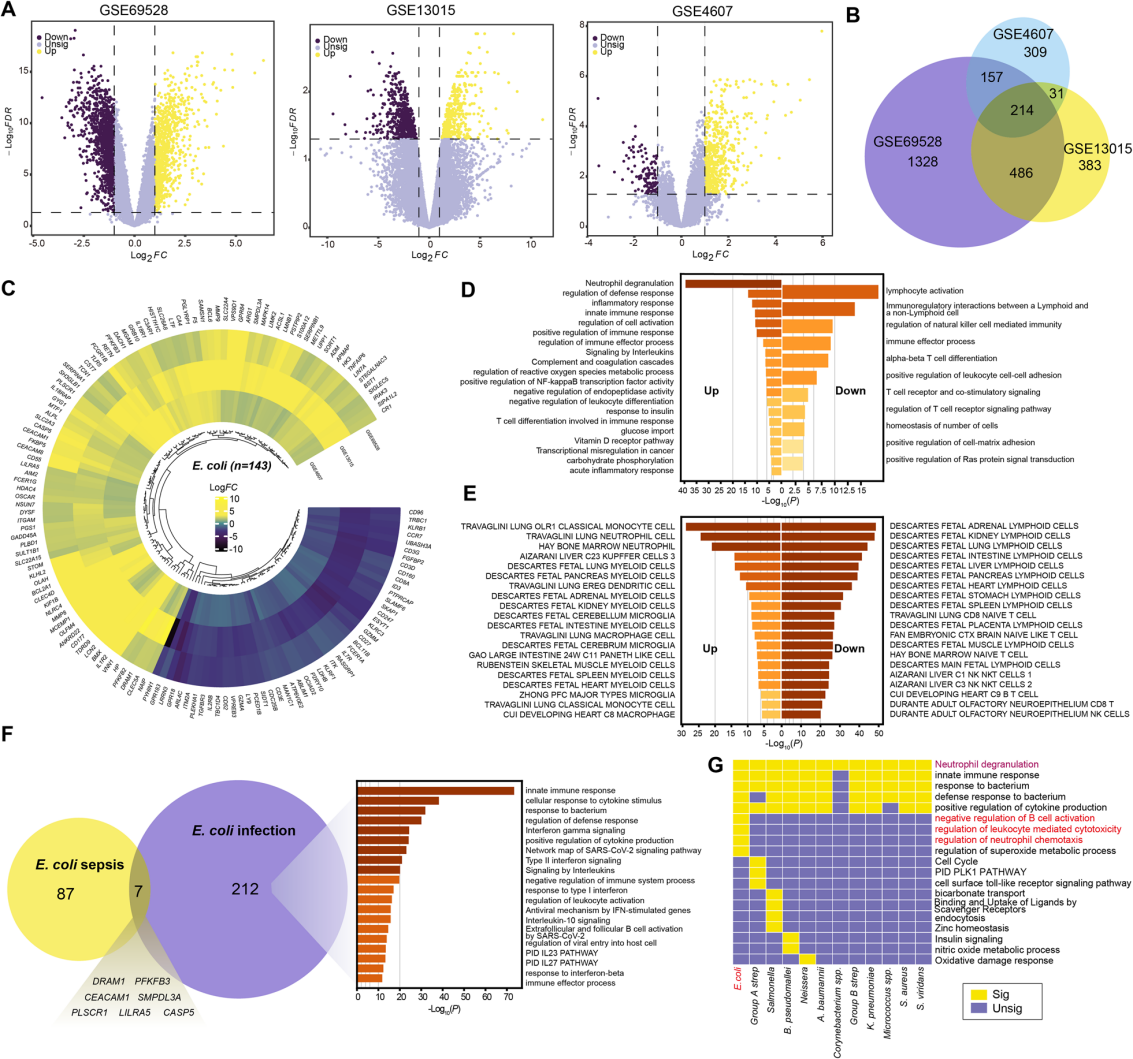
Dysregulated features of *E. coli* sepsis. **A** Volcano plots depict the DEGs between *E. coli* sepsis and controls in each bulk dataset. **B** The venn diagram illustrates the relationship of DEGs from various datasets. **C** The circle heatmap displays the expression of 143 underlying key genes of *E. coli* sepsis in each dataset. **D** and **E** Bar graph of enriched terms in biological function **D** and cell type signatures **E** across the 143 genes, colored by *Q* values. **F** The venn diagram (left) depicts the relationship of DEGs from ex vivo whole blood infection with *E. coli* and *E. coli* sepsis, a bar graph (right) shows the enriched terms across 212 genes, colored by *Q* values.** G** The heatmap shows the common and specific function terms across all types

In our study, we combined multiple types of omics data to investigate the cell-level heterogeneity in septic patients with 12 different bacterial infections (Fig. 1). To investigate the dysfunctional features of septic patients with different bacterial infections, we integrated three bulk transcriptome datasets (GSE69528, GSE13015, and GSE4607) to identify DEGs, and then performed functional enrichment analysis. These datasets provided detailed information about the infectious pathogens identified in patients. As for septic patients with *E. coli* infection (hereinafter referred to as *E. coli* sepsis), we identified 214 DEGs (*FDR* < 0.05 with |Log2*FC*|> 1) shared among three cohorts (Fig. 2A, B). To obtain more reliable key genes, we further screened these DEGs using the RRA package (*P* < 0.05, Freq = 3). Finally, 143 key genes underlying *E. coli* sepsis were identified, including 94 upregulated and 49 downregulated genes (Fig. 2C).

Next, we performed functional annotation of DEGs using the Metascape database, and found that the upregulated genes were mainly enriched in “Neutrophil degranulation” and “Inflammatory response”, while downregulated genes were mainly enriched in “Lymphocyte activation” and “Regulation of natural killer cell mediated immunity” (Fig. 2D). Cell type enrichment analysis further revealed that genes with high expression displayed myeloid cell features (including *CD177*, *LTF*, and *ITGAM*), while genes with low expression exhibited lymphoid cell features (including *CD3E*, *CD8A*, and *CD8B*) (Fig. 2E, Additional file 2: Figure S1A), which may reflect the depletion of lymphocytes in septic patients [40].

Intriguingly, marked differences in gene expression were detected between ex vivo whole blood infected by *E. coli* and blood from patients with *E. coli* sepsis. Notably, genes enriched in T cell activation were significantly upregulated in *E. coli* infection (Log(*Q*) = −20.663), but significantly downregulated in *E. coli* sepsis (Log(*Q*) = 13.992), indicating that T cells may play distinct roles in *E. coli* infection and *E. coli* sepsis. Additionally, we identified some genes shared between these two conditions, including *DRAM1*, *PFKFB3*, and *CASP5*, which are related to autophagy [41], glycolysis [42], and pyroptosis [43], respectively, suggesting that alterations in metabolic and immune response pathways in immune cells may be a fundamental feature of *E. coli* infection (Fig. 2F).

Regarding septic patients caused by other pathogens, analysis of publicly available data (Table 4) identified 12 potential key gene sets in septic patients caused by 12 different pathogen infections (Additional file 2: Figure S1B, C). Functional annotation of these genes showed some common terms in all types of sepsis (Additional file 2: Figure S1D, Additional file 1: Table S1), including “Neutrophil degranulation,” “Innate immune response,” and “Response to bacterium” (Fig. 2G). In addition, some terms were specific to certain types of sepsis. For example, “Negative regulation of B cell activation” was specific to *E. coli* sepsis, “Insulin signaling” was exclusively high in *B. pseudomallei* sepsis, and “Binding and uptake of ligands by scavenger receptors” was unique to *Salmonella* sepsis (Fig. 2G). Overall, we identified potential key genes and functional terms for each pathogen, indicating that septic patients with different bacterial infections both share common dysfunctional patterns and exhibit unique alterations.

**Table 4:** The number of DEGs in septic patients with different bacterial infections

Pathogens	Up	Down	Origin datasets
*A. baumannii*	55	12	GSE69528
*B. pseudomallei*	100	120	GSE69528, GSE13015
*Corynebacterium spp.*	17	26	GSE69528
*E. coli*	94	49	GSE69528, GSE13015, GSE4607
*Group A strep*	78	39	GSE4607, GSE69528
*Group B strep*	76	42	GSE4607
*K. pneumoniae*	43	22	GSE69528
*Micrococcus spp.*	55	10	GSE69528
*Neissera*	72	29	GSE4607
*Salmonella*	85	22	GSE69528
*S. aureus*	37	28	GSE4607, GSE69528
*S. viridans*	58	13	GSE4607

*Acinetobacter baumannii (A. baumannii), Corynebacterium species (Corynebacterium spp.), Group A Streptococcus (Group A strep), Group B Streptococcus (Group B strep), Klebsiella pneumoniae (K. pneumoniae), Micrococcus species (Micrococcus spp.), Staphylococcus aureus (S. aureus), Streptococcus viridans (S. viridans)*

### Identification of signature genes of sepsis with different bacterial infections

**Fig. 3:**
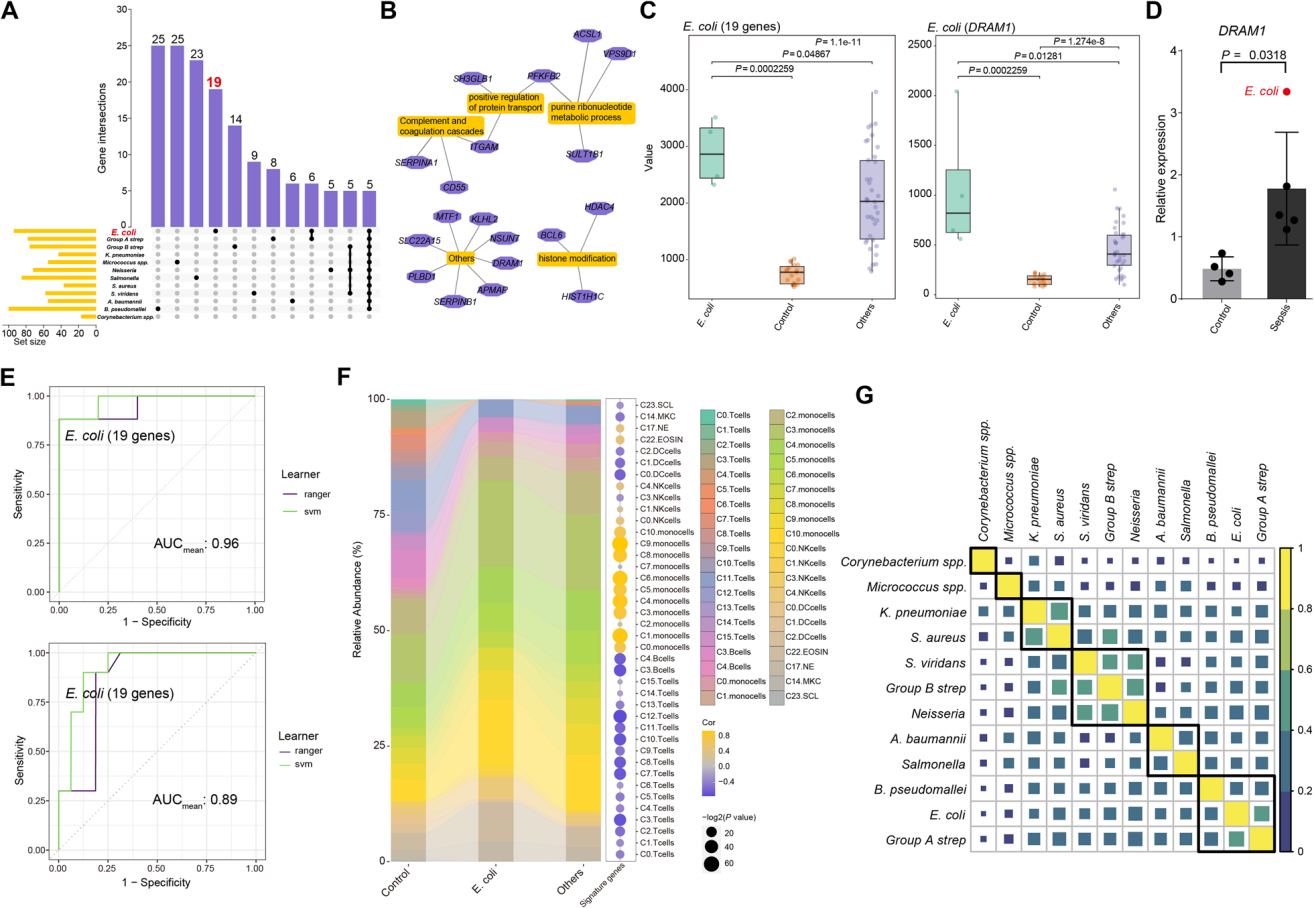
The signature genes of *E. coli* sepsis. **A** Upset plots show the unique and shared DEGs among different types of sepsis. **B** The signature genes of *E. coli* sepsis are identified. **C** The boxplot shows the average expression of signature genes and *DRAM1*. The *P* values are from a Wilcoxon test. **D** qRT-PCR analysis of DRAM1 expression in a cohort enrolled in this study (controls, n = 4; septic patients, n = 5). *P* value is determined by unpaired Welch's t-test. **E** Receiver operating curve for out-of-sample prediction of case–control state (up) and differentiation between *E. coli* sepsis and other types of sepsis (down) is trained on signature genes. **F** The Sankey plot (left) shows the cell abundance of each cluster (n = 40) across the 3 groups (controls, *E. coli* sepsis, and others). The heatmap (right) shows the correlation between signature genes and clusters. **G** The heatmap displays the similarity between different types of sepsis, with size and color indicating the similarity coefficient

We next sought to identify gene markers that can distinguish septic patients with different bacterial infections. By analyzing DEGs, we identified 19 genes that were exclusively upregulated in *E. coli* sepsis (Fig. 3A). These genes, which were mainly associated with complement activation, protein transport, purine metabolism, and epigenetic modification (Fig. 3B), are associated with clinical outcomes and disease progression in septic patients [44–46]. Of particular note, *DRAM1*, a signature DEG in *E. coli* infection (Fig. 2F), was identified as unique to *E. coli* sepsis (Fig. 3B), further emphasizing the critical role of *DRAM1* in *E. coli*-induced immune cell dysregulation. Moreover, we identified several DEGs in common among septic patients, including *VNN1*, *ANKRD22*, *TDRD9*, *S100A12*, and *MMP9* (Fig. 3A), which are primarily associated with neutrophil degranulation, suggesting a ubiquitous role for this process in septic patients.

We further evaluated the potential diagnostic role of the unique DEGs using an additional dataset (GSE13015_GPL6106), and found that expression of the 19 signature genes was higher in patients with *E. coli* sepsis than in healthy controls and septic patients caused by other bacteria (*P*_*sepsis* vs controls_ = 2.259e-04, *P*_*E. coli* vs Others_ = 0.04867) (Fig. 3C). We also validated that the expression of *DRAM1* was higher in septic patients than in controls in our enrolled cohort (*P* = 0.0318) (Fig. 3D). Furthermore, the 19-gene signature showed good ability to predict case–control status (AUC_mean_ = 0.96) (Fig. 3E). Further, the model showed a strong ability to distinguish *E. coli* infection from other bacterial infections (AUC_mean_ = 0.89) (Fig. 3E). These results highlight the effectiveness of our signature gene set for identifying and characterizing different types of sepsis.

We also analyzed other types of sepsis in the same way, and found that the signature gene set for *B. pseudomallei* was highly expressed in *B. pseudomallei* sepsis (Additional file 2: Figure S2A). Furthermore, the signature set had good predictive value for both case–control status (AUC_mean_ = 0.91) (Additional file 2: Figure S2B) and distinction between *B. pseudomallei* sepsis and other types of sepsis (AUC_mean_ = 0.90) (Additional file 2: Figure S2C). In addition, signature gene sets for other types of sepsis also showed high expression levels in their respective patient cohorts and demonstrated impressive classification performance (Additional file 2: Figure S2A, B). These results highlight the efficacy of our signature gene sets (Additional file 2: Table S2) for identifying and characterizing different types of sepsis.

Finally, we analyzed the relationships among different types of sepsis. Based on the Jaccard similarity coefficient, we found that *S. viridans* sepsis was similar to *Group B Streptococcus* (*Group B Strep*) sepsis, while the *E. coli* sepsis and *Group A Streptococcus* (*Group A Strep*) sepsis clustered together (Fig. 3G). Moreover, through deconvolution analysis, we observed several differences in cell clusters between *E. coli* sepsis and those of other type of sepsis, including C0.monocell (*P* = 0.043), C2.monocell (*P* = 0.009), and C7.monocell (*P* = 0.006) (Fig. 3F), suggesting that different types of sepsis may be associated with different cell clusters and emphasizing the need for further single-cell level analysis.

### Septic patients with different bacterial infections are distinguished with immune cell subclusters

As septic patients with different bacterial infections exhibited cell type-specific dysregulation, we attempted to identify specific cell clusters related to different bacterial infections by integrating three published scRNA datasets (GSE151263, GSE167363, and SCP548). After quality control of the single-cell transcriptome data (Additional file 2: Figure S3A), we obtained a large scRNA dataset comprising 152,636 cells from 69 samples (21 controls and 48 septic patients).

**Fig. 4:**
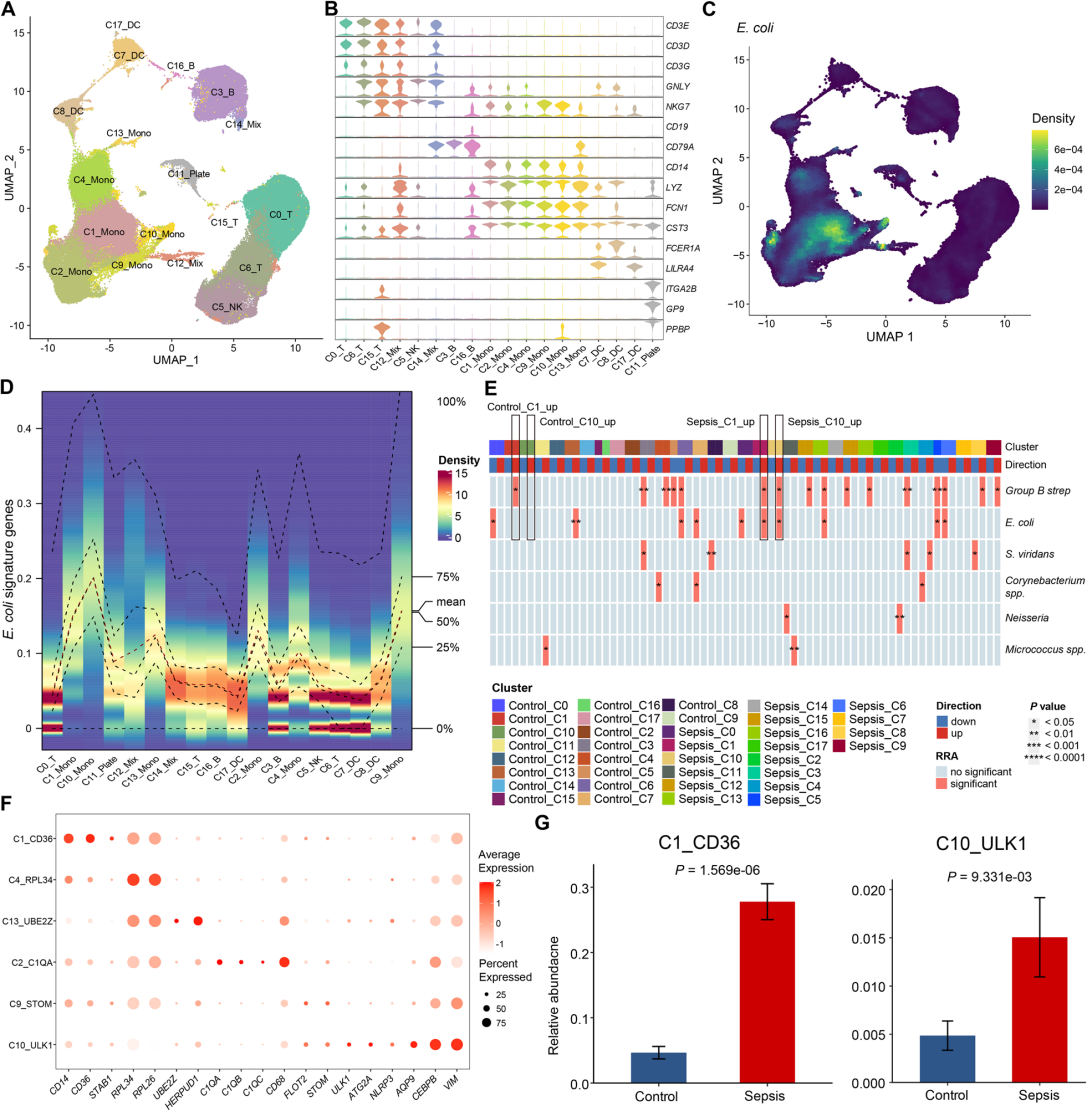
The related cell clusters of *E. coli* sepsis. **A** The UMAP plot shows the clustering of 152,636 cells from 69 samples into 18 clusters. **B** Cell clusters are defined by a set of known marker genes. **C** The density scatter plot shows the expression levels of the *E. coli* sepsis signature. The color gradient represents the enrichment score, yellow indicates a higher score. **D** The density heatmap shows the expression and distribution of the *E. coli* sepsis signature in different cell subclusters. The color gradient represents the enrichment score, red indicates a higher score. **E** The heatmap shows the statistical significance of the *E. coli* sepsis signature in each cell subcluster as determined by the RRA method. The top bar graph shows the different cell subclusters, and the bottom bar graph shows the upregulation or downregulation of the signature gene set in each subcluster. **F** The marker genes of each monocyte cluster. **G** The bar chart shows the relative cell abundance of C1_CD36 and C10_ULK1 in controls and septic patients. *P* values are from a Wilcoxon test

We then performed unsupervised clustering of the single-cell transcriptome data and identified 18 clusters, followed by two-dimensional uniform manifold approximation and projection (Fig. 4A, B). To investigate the specific clusters associated with patients with different types of sepsis, we calculated signature gene set scores for each cell using “irGSEA.” We found that the signature genes for *E. coli* sepsis were mainly enriched in monocytes (Fig. 4C, D). Further, the signature gene set for *E. coli* sepsis was significantly upregulated in patient C1_Mono and C10_Mono cell clusters (Fig. 4E), indicating that *E. coli* sepsis may be associated with dysfunction of these clusters. Subsequently, we renamed these monocyte cell clusters based on their distinctive marker genes (Fig. 4F). Notably, the C1_CD36 and C10_ULK1 clusters associated with *E. coli* sepsis were significantly over-represented in septic patients (Fig. 4G).

Regarding other types of sepsis, we found that the signature genes for *Group B strep*, *Neisseria*, and *S. viridans* sepsis were also mainly enriched in monocytes (Additional file 2: Figure S3B). However, unlike *E. coli* sepsis, signature genes for *S. viridans* sepsis were predominantly enriched in the C4_RPL34 cluster, while those for *Group B strep* sepsis were enriched in C13_UBE2Z cells. In addition, the signature gene set for *Micrococcus* spp. sepsis was enriched in platelets (Additional file 2: Figure S3B). Taken together, our data reveal the dysregulated cell clusters associated with different types of sepsis.

### Signature genes are related to the pathologic roles of characteristic cell clusters

**Fig. 5:**
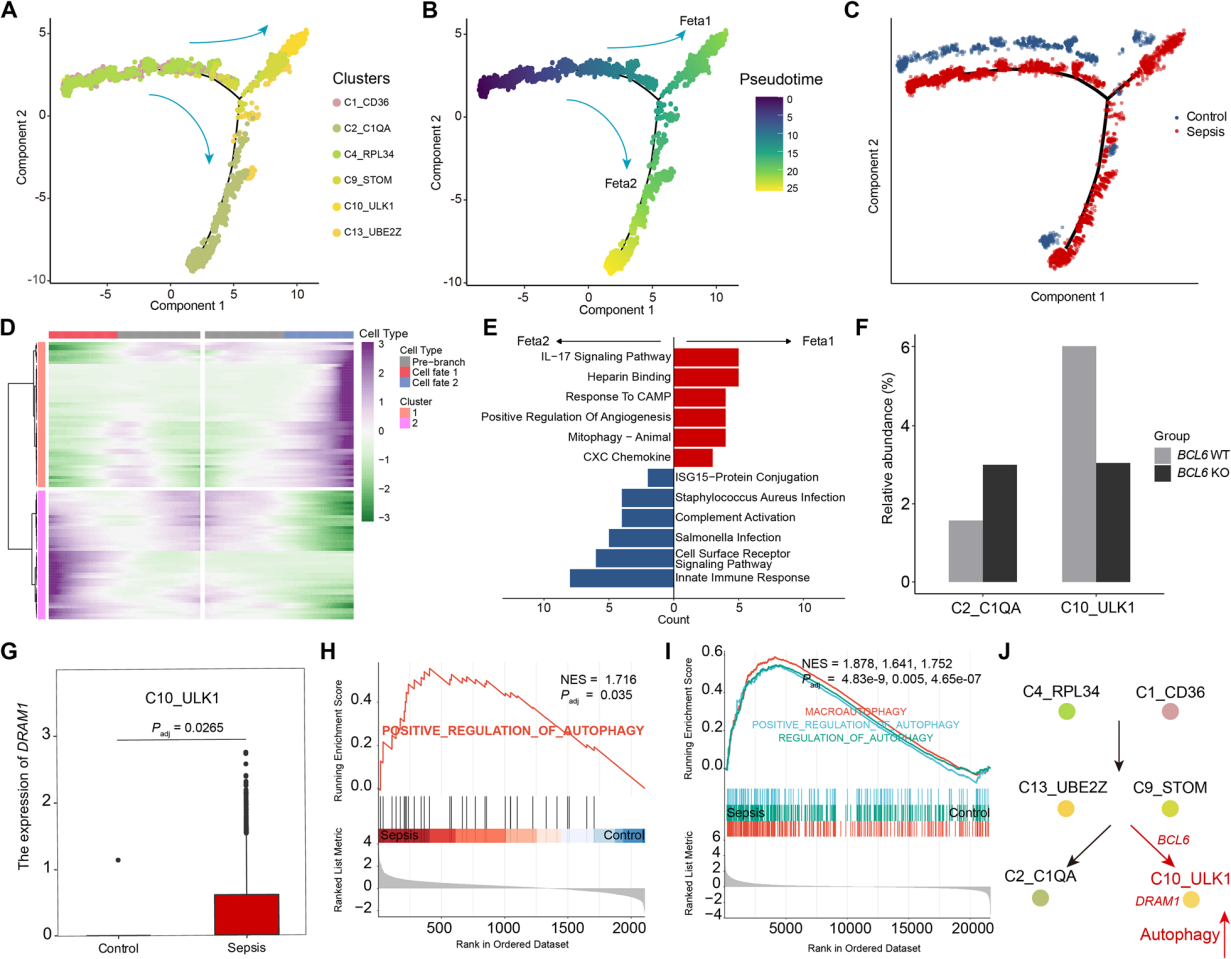
Function of signature genes of *E. coli* sepsis in related cell clusters. **A-C** Pseudotime analysis of monocyte cell clusters. Trajectory of monocyte cell clusters is inferred using monocle2 and clusters are marked by colors **A** Pseudotime-ordered variables are inferred **B** Cells derived from case or control are displayed separately on the differentiation trajectory **C** Lines and arrows indicate inferred differentiation trajectory and direction. **D** Heatmap showing the expression of genes related cell fate decision. **E** Functional enrichment of genes related to two differentiation trajectories. **F** The bar chart shows the relative cell abundance of C2_C1QA (left) and C10_ULK1 (right) in WT and KO mice. **G** Expression of *DRAM1* in C10_ULK1 between sepsis and controls. The *P* value is from a Wilcoxon test. **H and I** GSEA of autophagy-related genes in C10_ULK1 **H** and GSE4607 dataset **I**, comparing controls and septic patients. Nominal *P* value and the false-discovery rate (*FDR*) are indicated. **J** Schematic diagram of monocyte cluster differentiation

Monocytes are major contributors to sepsis pathogenesis [8, 47], and our study also found intimate associations between bacterial sepsis and monocytes. We next performed pseudotime analysis of six monocyte cell clusters using monocle2, to investigate the cell type-specific characteristics of monocytes associated with sepsis. We found that the C1_CD36 and C4_RPL34 clusters, located at the start of the trajectory, transitioned through C9_STOM and C13_UBE2Z to two terminal clusters, C2_C1QA, and C10_ULK1 (Fig. 5A). We defined the trajectory to C10_ULK1 as “Fate1,” and the trajectory to C2_C1QA as “Fate2” (Fig. 5B). Moreover, relative to controls, the early-stage monocyte cluster, C4_RPL34, was significantly reduced in sepsis, while the intermediate and late-stage monocyte clusters, C2_C1QA, C9_STOM, and C10_ULK1, were significantly expanded in sepsis (Fig. 5C, Additional file 2: Figure S4A). Next, we investigated the associated genes and pathways underlying the two differentiation trajectories (Fig. 5D). We identified that genes expressed in Fate1 were involved in “IL-17 Signaling Pathway,” “CXC Chemokine,” “Mitophagy—Animal,” “Positive Regulation of Angiogenesis,” and “Heparin Binding,” while those in Fate2 were associated with “Complement Activation” and “ISG15-Protein Conjugation” (Fig. 5E). We obtained similar results when comparing C2_C1QA and C10_ULK1 (Additional file 2: Figure S4B, C). Notably, in consistent with the observation shown in Additional file 2: Figure S3B, the genes highly expressed in C10_ULK1 were enriched in “Pathogenic *Escherichia coli* infection,” while those in C2_C1QA were enriched in “*Salmonella* infection” (Additional file 2: Figure S4C). These findings indicate that monocytes with different fates have unique functions and that specific clusters play crucial roles in specific types of sepsis.

Intriguingly, branching analysis indicated that *BCL6*, an *E. coli* sepsis signature gene, was associated with fate decision (*P*_adj_ = 2.3e-24). To further investigate the role of *BCL6* in *E. coli* sepsis, we performed a deconvolution analysis of a public dataset (GSE24813) from WT and *Bcl6*-KO mice. Our results showed that the C2_C1QA cell cluster was over-represented, while C10_ULK1 was under-represented in *Bcl6*-KO mice (Fig. 5F), suggesting that *Bcl6* may be important in monocyte differentiation. In addition, we found that *DRAM1* was significantly upregulated in C10_ULK1 from patients with *E. coli* sepsis (Fig. 5G). As *DRAM1* is a key regulator of autophagy [48], we investigated whether autophagy pathways were altered by gene set enrichment analysis, based on GO analysis, and found that functional terms related to autophagy were significantly upregulated in patient C10_ULK1 cluster cells (Fig. 5H). We also observed a significant upregulation of autophagy in patients with *E. coli* sepsis (GSE4607) (Fig. 5I). Therefore, we believed that the relationship between *DRAM1* and *E. coli* sepsis is related to the upregulation of autophagy in C10_ULK1.

Regarding other sepsis types, we found that *Neisseria* sepsis was significantly associated with C2_C1QA cluster cells. Additionally, *C1QA*, a *Neisseria* sepsis signature gene, was significantly upregulated in C2_C1QA cluster cells from septic patients (Additional file 2: Figure S4D). Furthermore, *S. viridans* sepsis was significantly associated with C4_RPL34, and *CTSD* may have an important role in this association (Additional file 2: Figure S4E). *Group B Strep* sepsis was significantly associated with multiple cell clusters, including C8_DC, C9_STOM, C10_ULK1, C12_mix, C13_UBE2Z, and C16_B, and its signature genes, including *CKAP4*, *CPEB4*, *TSPO*, and *TXN*, were significantly upregulated in multiple relevant clusters (Additional file 2: Figure S4F). In addition, although some types of sepsis did not show significant enrichment in relevant clusters after strict RRA correction, we found that they were associated with certain cell clusters and that their signature genes differed significantly in these clusters (Additional file 2: Figure S3B). For example, *Salmonella* sepsis was associated with the C2_C1QA cluster, and the differential signature genes were *MARCO* and *LILRB4* (Additional file 2: Figure S4G). *Micrococcus* spp. sepsis was associated with the C11_Plate cluster, and *ESAM*, *HBG2*, *MMRN1*, *PF4V1*, *SAMD14*, *SELP*, and *TGFB1I1* may be important in this context (Additional file 2: Figure S4H).

Taken together, these findings suggested that signature gene sets are important for the differentiation and dysfunction of related cell clusters. In particular, *DRAM1* and *BCL6* were implicated in autophagy and differentiation of C10_ULK1 cluster cells, respectively (Fig. 5J). Therefore, in sepsis caused by particular types of bacterial infections, targeting these specific genes could potentially serve as a stratified therapeutic strategy to enhance patient outcomes.

### DRAM1 promotes autophagy and glycolysis in an inflammatory monocyte subset in *E. coli* sepsis

**Fig. 6:**
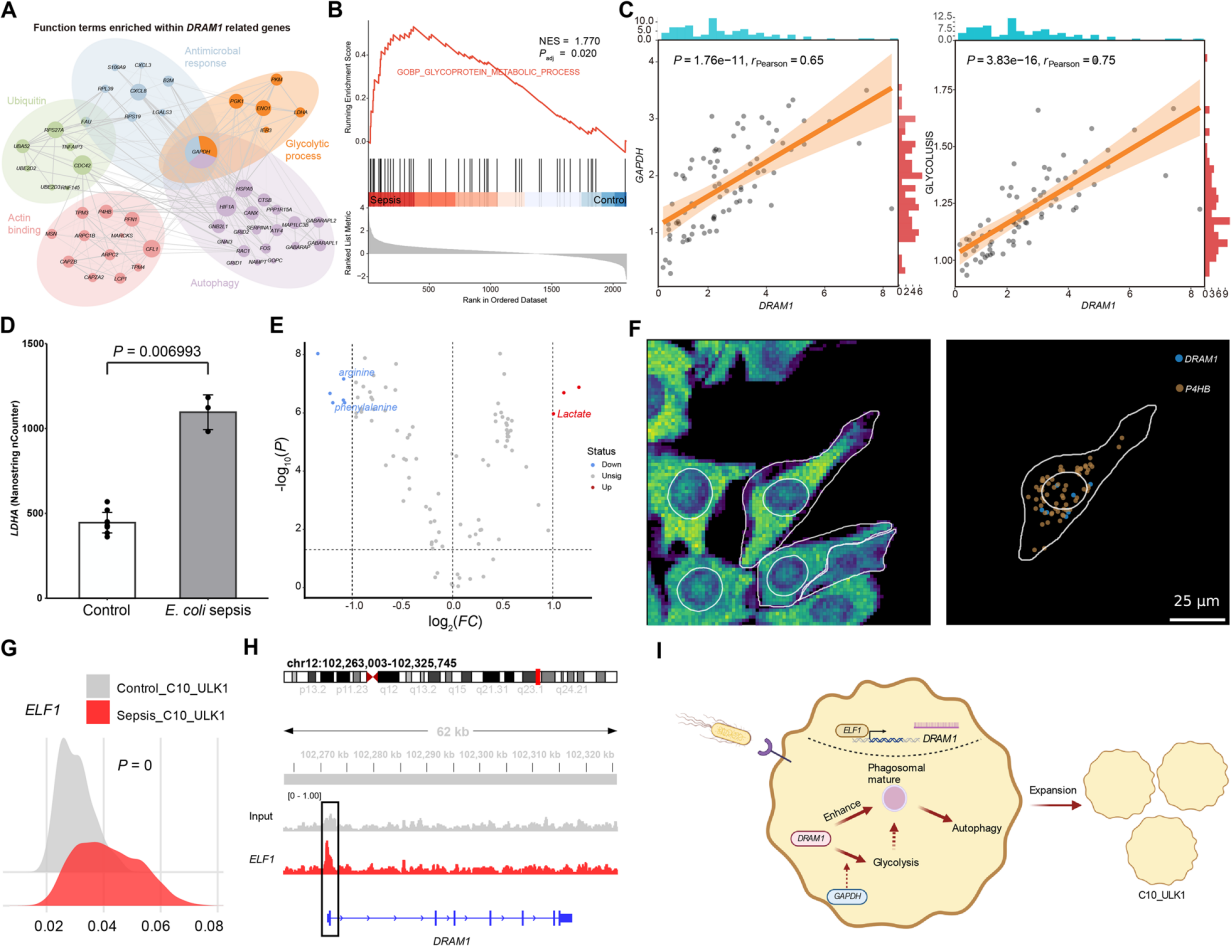
Function of DRAM1 in related cell clusters. **A** Functional annotation of DRAM1-related genes. **B** GSEA of glycolysis-related genes in C10_ULK1 comparing controls and septic patients. **C** Two-dimensional plots show the correlation between *DRAM1* and *GAPDH*, glycolysis-related genes (from “HALLMARK_GLYCOLYSIS”). **D** The bar chart shows the expression level of *LDHA* in controls and *E. coli* septic patients. **E** Volcano plots depict the differential metabolites between bacterial sepsis and controls. **F** The 2D image displays the RNA density (left) and the distributions of *DRAM1* and *P4HB* in cells (right). **G** The ridgeline plot shows the expression level of *ELF1* in C10_ULK1 between controls and septic patients. **H** *ELF1* ChIP-seq peaks at the promoter regions of *DRAM1*. **I** The schematic diagram shows the regulatory molecular mechanism of *DRAM1* in C10_ULK1 in *E. coli* sepsis

To reveal the potential mechanisms underlying cell function regulation by subset-related signature genes, we performed co-expression analysis in related cell clusters using GEN3. Genes co-expressed with *DRAM1* were enriched for “Autophagy,” “Glycolytic process,” “Antimicrobial responses,” “Ubiquitin,” and “Actin binding” functions (Fig. 6A). Further, we observed significant upregulation of glycoprotein metabolic process in C10_ULK1 cells (Fig. 6B). Interestingly, we found that *GAPDH*, which encodes a key enzyme in the glycolytic reaction, glyceraldehyde-3-phosphate dehydrogenase, was involved in autophagy regulation, which may also be related to *DRAM1* (Fig. 6A). To further investigate the relationships between *DRAM1* and *GAPDH*, as well as *DRAM1* and glycolysis, we conducted correlation analysis of a bulk RNA dataset from septic patients (GSE4607), which showed that *DRAM1* was significantly correlated with *GAPDH* (*P* = 1.76e-11, r = 0.65) and glycolysis (*P* = 3.83e-16, r = 0.76) (Fig. 6C). In addition, nCounter analysis of a gene expression dataset (GSE167914) related to cell metabolism identified *LDHA*, which encodes a key enzyme in the final step of glycolysis, as a *DRAM1*-coexpressed glycolytic gene (Fig. 6A) that was significantly upregulated in patients with *E. coli* sepsis (Fig. 6D). Consistently, *LDHA* expression was higher in septic patients than in controls in the enrolled cohort (*P* = 0.0169) (Additional file 2: Figure S5A). Metabolomics analysis also showed a significant increase in lactate levels in septic patients with bacterial infection (Fig. 6E) [21]. These results suggest that *DRAM1* may play an important role in *E. coli* sepsis by regulating autophagy and glycolysis in C10_ULK1 cluster cells.

To further explore the relationship between *DRAM1* and its co-expressed genes, we performed subcellular gene co-localization analysis to determine whether they tended to be transcribed in close proximity. By calculating the distance between genes, we found that *P4HB*, a gene associated with autophagy, co-localized with *DRAM1* (Fig. 6F). *DRAM1* was also in close proximity to *FAM49B* (related to bacterial infection [49]), *THBS1* (related to autophagy [50]), *TAGLN2* (related to phagocytosis [51]), and *PDIA3* (related to autophagy [52]) (Additional file 2: Figure S5B), which displayed higher expression levels in and around the cell nucleus (Additional file 2: Figure S5C). This further strengthens regulatory function of *DRAM1* in autophagy and bacterial infection.

Furthermore, we performed SCENIC analysis to identify upstream factors regulating *DRAM1* expression and found that *DRAM1* could be regulated by the transcription factor, *ELF1*. By analyzing the ChIP-seq dataset (GSE122203), we validated that ELF1 significantly bound to the *DRAM1* promoter region relative to an input control (Fig. 6H). Additionally, *ELF1* was significantly upregulated in C10_ULK1 cells from patients (Fig. 6G), indicating its potential role in response to bacterial infection. Together, our findings suggest that *DRAM1* induced by ELF1 could upregulate autophagy to resist bacterial infection and promote glycolysis (presumably through its association with *GAPDH*) for autophagic energy supply during *E. coli* infection (Fig. 6I).

Regarding other types of sepsis, co-expressed genes were mainly enriched for “Response to bacterium” and “Inflammatory response” (Additional file 2: Figure S5D-I). We found that *C1QA* may enhance phagocytosis and regulate immune interactions in C2_C1QA cells to participate in *Neisseria* sepsis [53] (Additional file 2: Figure S5D). Cell activation and cytokine production mediated by *MARCO* and *LILRB4* in C2_C1QA cells may be crucial in *Salmonella* sepsis (Additional file 2: Figure S5E, F). In C4_RPL34 cells in the context of *S. viridans* sepsis, genes co-expressed with *CTSD* were related to “Leukocyte migration,” “Intrinsic apoptotic regulation,” and “MYD88: MAL cascade initiation,” which may contribute to disease pathogenesis (Additional file 2: Figure S5G). Genes co-expressed with *CKAP4* in C12_mix, involved in “PID pathway,” “Regulation of peptidase activity,” and “Vitamin D receptor pathway,” and might contribute to regulation of Group B *Strep* sepsis (Additional file 2: Figure S5H). Notably, the close relationship in expression among *HBG2*, *MMRN1*, *SAMD14*, *SELP*, and *TGFB1I1* in C11_Plate cells suggests that they may participate together in *Micrococcus* spp. sepsis (Additional file 2: Figure S5I). Overall, our findings shed light on potential mechanisms involving key signature genes in related cell subpopulations, which could provide a theoretical foundation for clinical translation in the future.

### Sepsis with different bacterial infections involves distinct cell communication networks in PBMCs

**Fig. 7:**
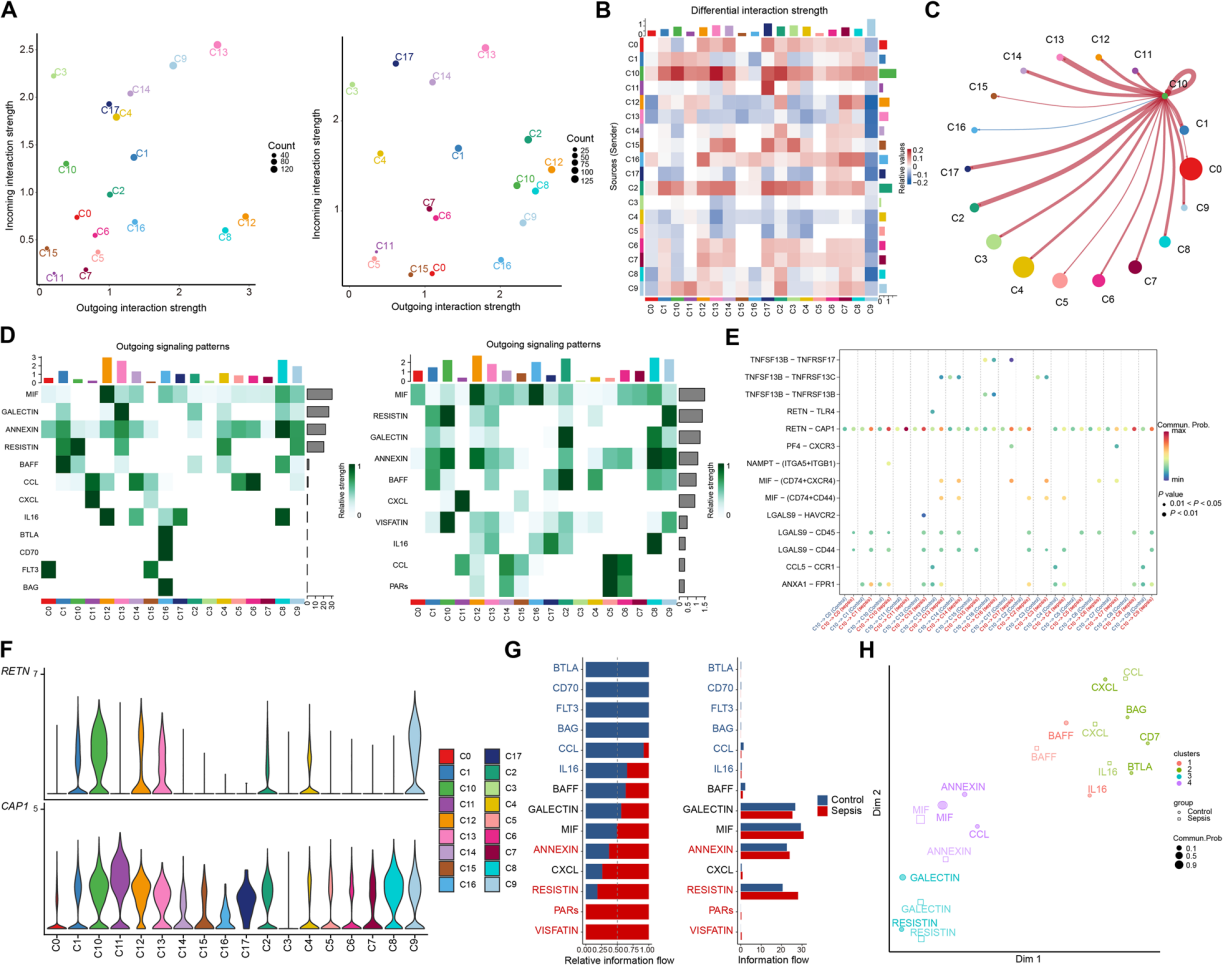
The role of relevant cell clusters in sepsis PBMCs. **A** The strength of outgoing and incoming signals of cell clusters in controls (left) and septic patients (right). **B** Heatmap of differential interactions of clusters in the cell–cell communication network. The top bar indicates the sum of incoming signaling, and the right bar indicates the sum of outgoing signaling. **C** Differential interaction strength between C10_ULK1 (outgoing) and other cell clusters (incoming). **D** Heatmap showing the relative importance of outgoing signaling in each cell group in controls (left) and septic patients (right). **E** Comparison of significant ligand-receptor pairs between controls and septic patients. **F** The expressions of *RETN* (up) and *CAP1* (down) in different cell clusters. **G** All the signaling pathways of controls and sepsis samples are presented in relative information flow (left) and overall information flow (right). **H** Two-dimensional manifold projection of signaling pathways based on their functional similarity

To investigate the contribution of related cells to sepsis, we analyzed cell–cell interactions in the integrated scRNA dataset. We found that C10_ULK1 cells had significantly upregulated outgoing interaction strength (Fig. 7A) and mainly interacted with C2_C1QA, C10_ULK1, C13_UBE2Z, and C17_DC cell clusters (Fig. 7B, C). Signal changes among cell clusters revealed that the “RESISTIN,” “ANNEXIN,” and “VISFATIN” pathways were upregulated in C10_ULK1 cells from septic patients (Fig. 7D). In particular, the “RETN-CAP1” signaling pathway was markedly induced in C10_ULK1 cells, and involved in interactions with other cell clusters (Fig. 7E). We also found that *RETN* was primarily expressed in C10_ULK1 and C9_STOM cells, while *CAP1* was expressed in numerous clusters (Fig. 7F). *CAP1* is the canonical receptor for resistin, which can induce inflammation in humans [54]. Further, both MIF-(CD74 + CXCR4) and MIF-(CD74 + CD44) were stimulated during interactions between C10_ULK1 and C3_B, C13_UBE2Z, C14_Mix (Fig. 7E). MIF is a key molecule closely associated with bacterial infection and the subsequent immune response [55]. Studies have shown that *MIF* participates in the occurrence and development of sepsis by recruiting inflammatory cells through receptor binding [56]. Our results suggest that MIF may exert its effects by binding to various receptors on different cell types in sepsis. In conclusion, our data indicate that C10_ULK1 cells contribute to systemic inflammation in *E. coli* sepsis, mainly by secreting the RETN and MIF cytokines.

We also observed cell–cell interaction changes in sepsis in other clusters. Both ingoing and outgoing interaction strengths of C2_C1QA cells were increased in sepsis (Fig. 7A), with MIF-(CD74-CD44) displaying the most significant increase (Additional file 2: Figure S6A, B). For C12_mix, we observed an increase in ingoing interaction strength, mainly related to the chemokines, CXCR3 and CXCR4 (Additional file 2: Figure S6C). In the disease state, the outgoing interaction strength of C9_STOM increased, with RETN-CAP1 a major upregulated signal (Additional file 2: Figure S6D). C11_Plate had significantly upregulated outgoing interaction strength and mainly interacted with C3_B, C14_Mix, and C17_DC (Additional file 2: Figure S6E). The major upregulated ligand-receptor pair between C11_Plate and DC cells was PF4-CXCR3 (Additional file 2: Figure S6F). Platelet factor 4 (*PF4*) is a small cytokine that regulates inflammation by binding to the CXCR3 receptor [57]. Overall, our findings suggest that interactions among these clusters may contribute to the systemic inflammatory response observed in sepsis.

Next, we evaluated key signals and found that the ANNEXIN, RESISTIN, PARs, and VISFATIN pathways were upregulated in septic patients (Fig. 7G). Additionally, based on functional similarity analysis, we found significant functional differences in the signaling pathways differentially enriched under healthy and disease conditions (Fig. 7H). We also identified cell communication patterns related to sepsis. Under normal conditions, the incoming signaling of C1_CD36, C4_RPL34, C9_STOM, and C10_ULK1 was characterized by “pattern 4” containing ANNEXIN and VISFATIN pathways. While under disease conditions, C2_C1QA incoming signaling was also characterized by the same pattern shared with the aforementioned four cell clusters (Additional file 2: Figure S6G). As for the outgoing signaling, nearly most cell clusters were dominated by the separated patterns in health controls, while these signals of cell clusters tended to be concentrated (Additional file 2: Figure S6H). For example, the outgoing signaling of C1_CD36, C3_B, C9_STOM, and C10_ULK1 cells was dominated by “pattern 1,” which included RESISTIN and VISFATIN in sepsis (Additional file 2: Figure S6H). These dynamic signaling patterns in health and disease indicate that disease-specific signaling activation is cell-type specific. In summary, our findings reveal interaction networks among different cell clusters under disease conditions, and identify the main communication signaling pathways for each cluster, providing a functional basis to better understand the roles of various immune cells in different types of sepsis.

### Sepsis with different bacterial infections involves distinct cell communication networks in the tissue microenvironment

**Fig. 8:**
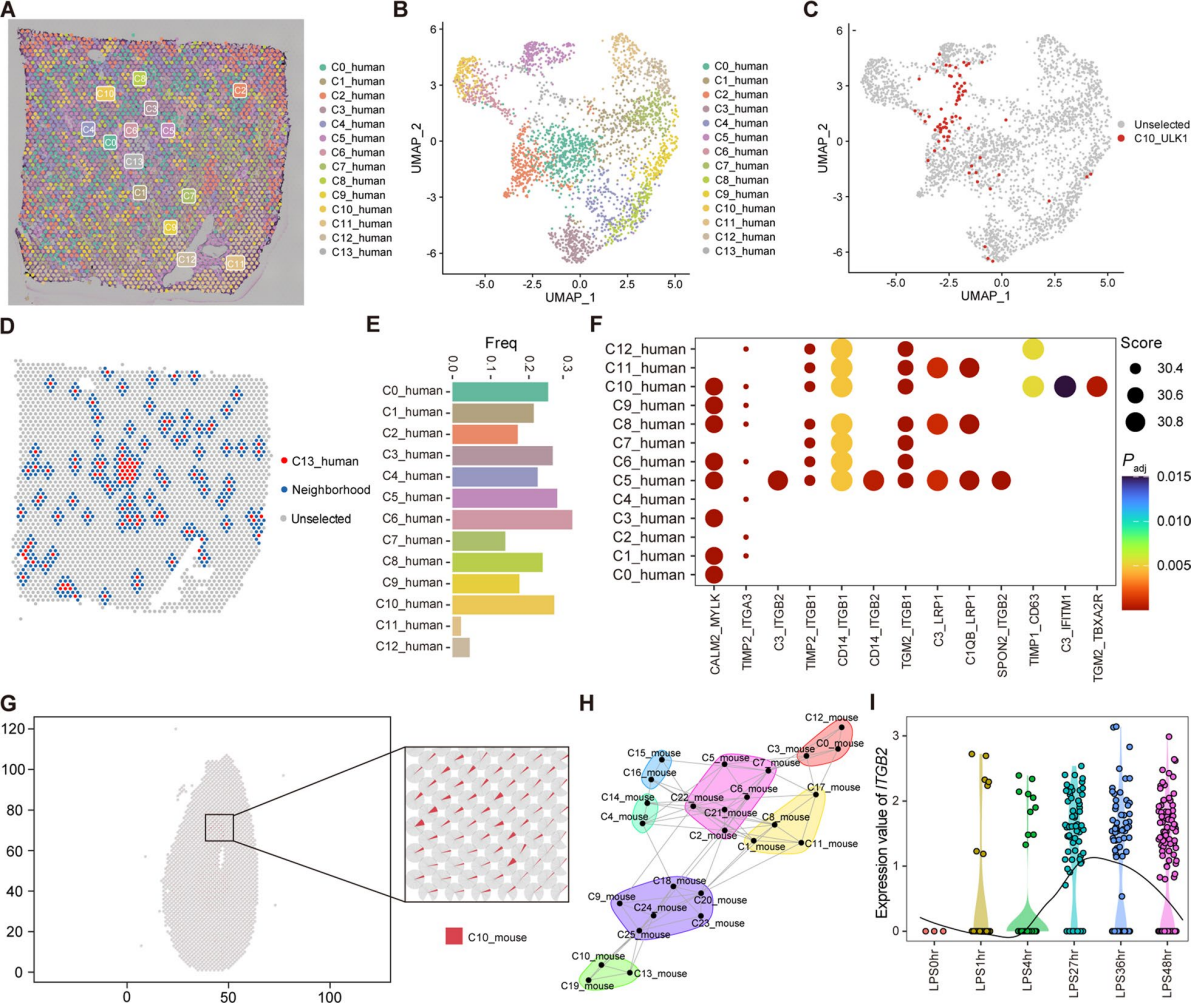
The role of relevant cell clusters in sepsis kidney tissue. **A** and **B** ST data from a human kidney tissue sample was processed and displayed with tissue sections **A** and UMAP plot **B**. Different colors represent different clusters. **C** The UMAP plot displays spots that were predicted to be C10_ULK1 in the spatial data. **D** The image depicts C13_human and its adjacent spots. **E** The image summarizes the proportions of different clusters present in the neighboring cells of C13_human. **F** The identified ligand-receptor pairs between C13_human and other clusters. **G** Localization of C10_mouse in kidney tissue. **H** Network community plot represents the communities of cells clusters from intrinsic view. **I** Expression of *ITGB2* at different time points after LPS injection in C19_mouse

Acute kidney injury is a common complication of sepsis that can lead to multi-organ dysfunction through its distant effects [58]. Therefore, we also examined cell interactions in kidney tissue by integrating scRNA and ST data. We analyzed an ST dataset from human kidney tissue (GSE171406) and identified 14 clusters (Fig. 8A). Next, we determined that C10_ULK1 was mainly enriched in cluster C13_human of ST, by mapping the PBMC clusters from the scRNA dataset onto the ST data (Fig. 8B, C). In kidney, podocytes (C6_human) were the most abundant neighboring cells (Fig. 8D, E). In addition, we found that integrin was a significant signal involved in interaction between C13_human and other cell clusters using SpaGene (Fig. 8F). Renal tubular obstruction in sepsis is primarily related to the shedding of proximal tubular epithelial cells, and is regulated by integrins [59]. Therefore, we speculated that C10_ULK1 may be involved in the pathogenesis of sepsis by regulating integrin signaling in kidney tissue.

To test this hypothesis, we further analyzed time-dependent scRNA and ST data from mouse sepsis model kidney tissues. First, we performed clustering analysis on the integrated mouse scRNA data and identified 26 cell clusters, of which C10_mouse displayed similar expression features to C10_ULK1 (Additional file 2: Figure S7A, B). To investigate spatial cell–cell interactions, we used the STRIDE method [33] to integrate scRNA and ST data (Fig. 8G, Additional file 2: Figure S7C). Based on the integrated data, we established models of intercellular interactions from different spatial perspectives (intraview, juxtaview, and paraview) using MISTy. By analyzing the contributions of each perspective to the prediction, we found that intraview generated a significant contribution (Additional file 2: Figure S7D). Next, we extracted interaction communities from intraview, and observed that C10_mouse cells closely interacted with the C19_mouse and C13_mouse clusters (Fig. 8H). Meanwhile, expression of *ITGB2* (encoding integrin beta) in C19_mouse was significantly increased under LPS stimulation (Fig. 8I). Therefore, integrin would be important for cell-to-cell interaction in kidney tissue in sepsis.

Furthermore, we found that the C2_C1QA cluster was mainly associated with C2_human cluster cells in human kidney tissue (Additional file 2: Figure S7E). Further analysis revealed that the C2_human cluster may function by secreting growth factors, such as VEGFA (Additional file 2: Figure S7F). High VEGFA expression in macrophages increases vascular permeability, leading to septic death. In contrast, inhibiting VEGF signaling can effectively reduce inflammation and protect mice with sepsis from death [60]. In conclusion, our study reveals that macrophages in kidney tissue may contribute to sepsis pathology by secreting growth factors and via integrin-mediated signaling.

### Virtual screening and de novo design reveal a potential strategy for stratified targeted therapy

**Fig. 9:**
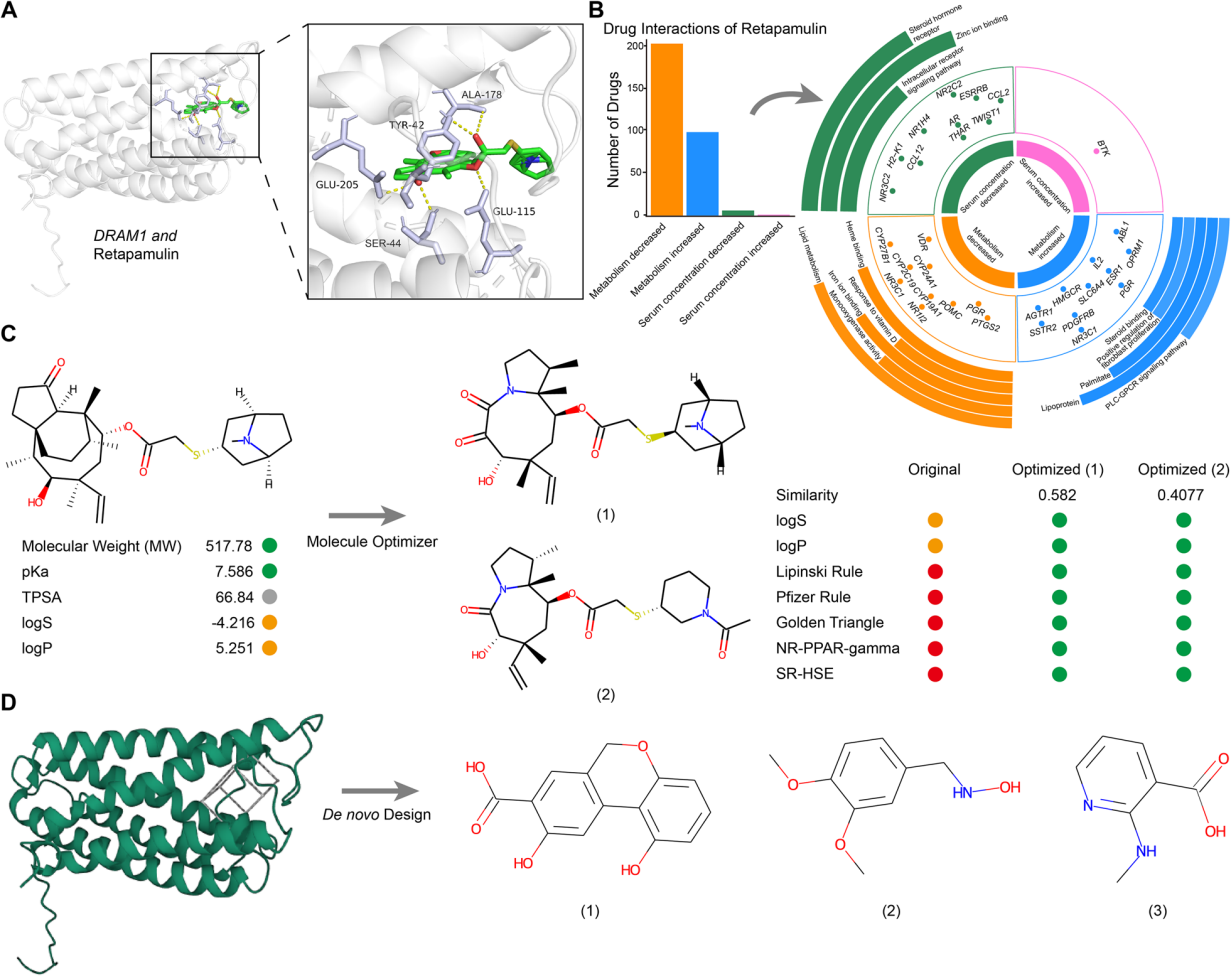
Identification of potential target drugs and molecules. **A** (Left) Protein–ligand docking complex of *DRAM1* with Retapamulin. (Right) Zoomed-in views of the interaction contact region. **B** (Left) Bar graph showing the number of drugs interacting with Retapamulin. (Right) Multi-ring chart showing different interaction effects, interaction drug targets, and target-enriched pathways from inner to outer rings.** C** Optimized molecular structure of Retapamulin and the associated improved properties.** D** De novo molecular design based on *DRAM1*

Given the crucial role of signature genes in definition of sepsis types, we explored the possibility for stratified therapy by targeting these genes with specific drugs by screening 5903 FDA-approved drugs from the ZINC20 database. For *E. coli* sepsis, we chose *DRAM1* for drug screening. After batching docking with Vina, to compute docking scores between *DRAM1* and each drug, 10 drugs were obtained. Among them, retapamulin exhibited the strongest affinity for *DRAM1* (Fig. 9A). Retapamulin is a topical antibiotic known to bind to *S. aureus* and *E. coli* ribosomes [61]. Further, we analyzed various drugs that can affect the pharmacological activity of retapamulin (Fig. 9B) [62], and found that its serum concentration can be increased by combination with abametapir, which is used to treat parasitic infections [63]. When used concomitantly with simeprevir [64] or boceprevir [65], (both treatments for chronic *hepatitis C virus* infection) retapamulin metabolism can be reduced. Conversely, concomitant use with rifampicin (for treating mycobacterial infections [66]) could enhance retapamulin metabolism, indicating that rifampicin would reduce the treatment duration. These findings highlight the importance of considering potential drug interactions when using retapamulin to treat *E. coli* sepsis in patients with complex clinical infections.

As retapamulin is a topical medication, we attempted to optimize its structure to assess its viability as an oral drug. After optimization, we identified two novel molecules exhibiting superior properties (Fig. 9C), which demonstrated enhanced potential for oral administration and exhibited favorable pharmacokinetic characteristics, indicating that they could be promising candidates for treatment of *E. coli* sepsis. Furthermore, our molecular design, based on protein pocket analysis, revealed potential molecules for targeted drug development, providing direction for further exploration and validation (Fig. 9D). For other types of sepsis, we also identified potential targeted drugs and small molecules, such as nystatiin-*C1QA* foe *Neisseria* sepsis and dalfopristin-*CTSD* for *S. viridans* sepsis (Additional file 2: Figure S8).

Overall, these drugs and molecules show great potential for use in tackling the challenges of stratified targeted therapy for sepsis with different bacterial pathogens, laying the groundwork for more effective and tailored therapeutic interventions.

## Discussion

Sepsis is a potentially fatal organ dysfunction that threatens tens of millions of people around the world, posing a significant global health challenge [1]. Bacterial infection-induced sepsis is the most common type, contributing to high morbidity and mortality rates [3]. Pathological mechanisms, functional impairments, clinical features, and treatment approaches generally differ among patients with distinct bacterial infections [4]. Therefore, comprehensive investigation of the heterogeneity among septic patients with different bacterial infections is necessary. In this study, we identified signature genes and relevant cell populations in patients with different types of sepsis. Furthermore, we determined the regulatory mechanisms in sepsis-related cells and explored the functions and signaling pathways in cell populations altered in the disease.

*E. coli* infection is a common cause of sepsis. However, comprehensive comparisons between *E. coli*-induced sepsis and sepsis with other bacterial infections are lacking. Through analysis of transcriptome data from 12 different types of bacteria-induced sepsis, we identified 19 signature genes unique to *E. coli* sepsis, most of which have established roles in sepsis and *E. coli* infection. For example, *ACSL1*, which is involved in shuttling fatty acids to mitochondria for β-oxidation and lipid synthesis pathways, is upregulated in human neonatal sepsis [44]. Further, *ACSL1* levels in macrophages increase in response to *E. coli* treatment [67]. However, the mechanism underlying the effects of *ACSL1*-mediated lipid metabolism in *E. coli* sepsis requires further investigation. Histone deacetylase 4 (*HDAC4*), which can be upregulated and activated by LPS [68], participates in septic myocardial injury by regulating HIF-1α acetylation [45]. Expression levels of the cell cycle-related gene, *HIST1H1C*, correlate directly with SOFA score and mortality rate [69] and *HIST1H1C* protein level is significantly upregulated following *E. coli* infection [70]. Although our study mainly focused on the role of *DRAM1* in *E. coli* sepsis, other signature genes might also have important roles and their molecular mechanisms require further exploration.

*DRAM1* was previously revealed to regulate autophagy against bacterial infection in macrophages [71]. Consistently, our findings demonstrate that upregulation of *DRAM1* was involved in regulating autophagy and glycolysis in C10_ULK1 cells in response to both *E. coli* infection and *E. coli* sepsis. We also discovered that *DRAM1* is co-expressed with *GAPDH*, a gene encoding a key enzyme in the glycolytic pathway that regulates autophagy in response to bacterial infection [72]. Similarly, fructose and mannose metabolism are significantly upregulated in *E. coli* sepsis, indicating a close relationship between glycolysis and *E. coli* sepsis [6]. Our findings suggest a potential crosstalk between *DRAM1*-regulated glycolysis and *GAPDH*-regulated autophagy. We speculate that activation of *DRAM1* may upregulate autophagy to counter bacterial infection, leading to expansion of C10_ULK1 cells in sepsis. Given the potential crosstalk between *DRAM1* and *GAPDH*, further exploration of *DRAM1* and *GAPDH* regulatory mechanisms in *E. coli* sepsis would be intriguing. Additionally, by high throughput drug screening, we identified retapamulin as a specific chemical targeting *DRAM1*. Retapamulin is an established antibiotic that selectively inhibits bacterial protein synthesis. Therefore, our new finding strengthens the prospect of clinical application of optimized retapamulin for treatment of *E. coli* sepsis.

Recently, monocytes have been revealed to be heterogenous in sepsis. For example, a distinct CD14^+^ monocyte cluster is expanded in bacterial sepsis and can function as a marker for distinguishing case–control states [8]. Based on single-cell transcriptome sequencing of PBMCs from controls and septic patients, NEAT1^+^ CD163^+^, and CD16^+^ monocyte clusters were identified as highly correlated with clinical indicators of sepsis [73]. In our research, we found that the C10_ULK1 monocyte subset was strongly associated with *E. coli* sepsis. This monocyte subcluster is at a late stage of cell differentiation, expresses high levels of autophagy- and inflammatory macrophage-related genes, and is significantly expanded in sepsis. Cell interaction analysis in PBMCs and kidney tissue revealed that C10_ULK1 can activate inflammatory signaling in different cells via secreted factors and cell–cell contact, leading to a systemic inflammatory response and contributing to *E. coli* sepsis occurrence and development. The role and function of this monocyte cluster in other diseases warrants further investigation.

Our study also identified signature genes, related cell clusters, and their interactions for each type of sepsis, where the signature genes showed close relationships with sepsis. For example, *MARCO*, the signature gene for *Salmonella* sepsis is highly expressed in macrophages from septic patients [74], while *SELP*, a *Micrococcus* spp. sepsis signature gene, was upregulated in platelets with increased mean platelet volume, leading to increased expression of P-selectin in sepsis [75]. Regarding related cell clusters, we found that most types of sepsis were associated with monocytes; *Neisseria* and *Salmonella* sepsis were related to C2_C1QA cells, while *S. viridans* sepsis was associated with C4_RPL34 cells. Additionally, we found that *Micrococcus* spp. sepsis was related to the C11_Plate cluster. Cell interaction analysis revealed changes in cell communication between different clusters in sepsis. For example, MIF-(CD74 + CD44) played an important role in cell communication in C2_C1QA cells, while C11_Plate may promote inflammation by regulating dendritic cells through PF4-CXCR3. Overall, our findings provide a basis for understanding the cellular heterogeneity among patients with different types of sepsis, but the underlying mechanisms and connections require further exploration.

In recent years, various transcriptome sequencing technologies and multi-omics analyses have been widely applied and facilitated great progress in the fields of cancer and developmental biology [76, 77]. However, the limited data from single-cell transcriptome analysis means that it is particularly important to integrate multi-omics data for systemic analysis in studies of sepsis. In our study, we identified marker genes for various sepsis subtypes by analyzing an integrated bulk RNA dataset. In addition, by investigating the enrichment of marker genes at the single-cell level, we identified cell clusters associated with different types of sepsis. Moreover, by combining multi-omics data, we revealed the regulatory mechanisms of signature genes in functions of specific cell populations. Finally, by combining human and mouse ST data, we were able to analyze the functions of relevant cell clusters in sepsis. Therefore, our study provides a more comprehensive and systematic understanding of the cellular heterogeneity in different types of sepsis, which has the potential to benefit patients. One limitation of our study is the relatively limited experimental validation. For holistic understanding and clinical application for septic patients, more comprehensive sequencing data and thorough studies will be imperative.

## Conclusions

By integrating multi-omics data, we have uncovered the cellular level heterogeneity of sepsis with different bacterial infections. We demonstrate that different types of sepsis are characterized by specific signature genes, cell populations, molecular mechanisms, cell functions, and underlying target drugs, which has important implications for the early diagnosis and stratified treatment of sepsis.

### Supplementary Information

**Additional file 1: Table S1.** Functional annotation of the potential key gene sets in septic patients with different bacterial infections.

**Additional file 2: Table S2.** The unique signature genes in septic patients with different bacterial infections. **Figure S1.** Dysregulated functions of other types. **A** Protein–protein interaction networks among the 143 genes. **B and C** The potential key gene sets identified from two datasets** B** and one dataset **C. D** Heatmap shows the function terms that were enriched in different types (Top 20 terms). **Figure S2.** The signature genes of other types. **A** Boxplots show the average expression of signature gene sets. *P* values are from a Wilcoxon test. **B and C** Receiver operating curves for out-of-sample prediction of case–control state **B** and differentiation between *B. pseudomallei* sepsis and others **C** trained on signature gene sets. **Figure S3.** The related cell clusters of other types. **A** The UMAP plot shows the origin of cells before (left) and after batch-correction (right) (harmony). **B** The density scatter plot shows the expression levels of all the signatures. The color gradient represents the enrichment score, with yellow indicating a higher score. **Figure S4.** The signature genes for other types of sepsis in related cell clusters. **A** The bar chart shows the relative cell abundance of C2_C1QA, C4_RPL34, C9_STOM and C13_ZNF703 in controls and septic patients. **B** Volcano plots depict the DEGs between C2_C1QA and C10_ULK1. **c** Functional enrichment of the DEGs between C2_C1QA and C10_ULK1. **D-G** Violin plots show the expression of signature genes in different clusters between sepsis and controls. **H** The dot plot shows the expression of signature genes of *Micrococcus spp.* sepsis in each cluster between sepsis and controls. *P* value is from a Wilcoxon test. **Figure S5.** Function of signature genes in related cell clusters. **A** qRT-PCR analysis of LDHA expression in a cohort enrolled in this study (controls, n = 4; sepsis patients, n = 5). *P* value is determined by unpaired Student's t-test.** B** The 2D image displays the RNA density (top) and the distributions of *DRAM1* and its co-expressed genes (*FAM49B*, *THBS1*, *TAGLN2*, and *PDIA3*) (bottom).** C** The density distribution plot illustrates the regions where *DRAM1* and its adjacent five genes are distributed within the cells**. D-H** Network of enriched terms by co-expressed genes of signature genes in related clusters. **I** *HBG2*, *MMRN1*, *SAMD14*, *SELP*, and *TGFB1I1* are involved in sepsis-related functions in C11_plate. **Figure S6.** Cell–cell communication among cell clusters in PBMCs. **A-D and F** Comparison of significant ligand-receptor pairs was conducted between C2_C1QA (outgoing) and other clusters (incoming) **A**, between other clusters (outgoing) and C2_C1QA (incoming) **B**, between other clusters (outgoing) and C12_mix (incoming) **C**, between C9_STOM (outgoing) and other clusters (incoming) **D**, and between C11_plate (outgoing) and other clusters (incoming) **F**, in both controls and septic patients. **e** Differential interaction strength between C11_plate and other cell clusters. **G and H** Inferred incoming and outgoing communication patterns of secreting cells between controls and septic patients. The inferred potential patterns are correspondent to cell populations and signaling pathways. The thickness of the flow indicates the contribution of the cell group or signaling pathway to each potential pattern. **Figure S7.** Cell–cell communication among cell clusters in kidney tissue. **A** The UMAP plot shows scRNA data from kidney tissue of control mice and sepsis model mice. **B** The UMAP plots show the marker genes of macrophage and C10_ULK1 in mouse kidney tissue. **C** The scatter pie plot shows the spatial locations of different cell types predicted by STRIDE. Colors represent different cell types. **D** The relative contribution of each view to the prediction of cell interactions. **E** The UMAP plot displays spots that are predicted to be C2_C1QA in the spatial data of human kidney tissue. **F** The identified ligand-receptor pairs between C2_human and other clusters. **Figure S8. Identification of potential target drugs and molecules. (A, C, E, G, and I)** (Left) Protein–ligand docking complex. (Right) Zoomed-in views of the interaction contact region. **(B, D, F, H, and J)** De novo molecular design based on C1QA **(B)**, MARCO **(D)**, LILRB4 **(F)**, CTSD **(H)**, or CKAP4 **(J)**.

## Data Availability

All data generated or analyzed during this study are included in this published article and its supplementary information files.

## Abbreviations

*A. baumannii*: *Acinetobacter baumannii*
*B. pseudomallei*: *Burkholderia pseudomallei*
ChIP-seq: Chromatin immunoprecipitation sequencing
ComICS: Computational methods for immune cell-type subsets
*Corynebacterium spp.*: *Corynebacterium species*
CRP: C-reactive protein
CT: Cycle threshold
DAVID: Database for annotation, visualization and integrated discovery
DC: Dendritic cell
DEGs: Differentially expressed genes
*E. col*: *Escherichia coli*
GEO: Gene expression omnibus
GO: Gene ontology
*Group A strep*: *Group A Streptococcus*
*Group B strep*: *Group B Streptococcus*
GSEA: Gene Set Enrichment Analysis
*HDAC4*: Histone deacetyltransferase 4
IGV: Integrative genomics viewer
KO: Knockout
*K. pneumoniae*: *Klebsiella pneumoniae*
*Micrococcus spp.*: *Micrococcus species*
MISTy: Multiview intercellular SpaTial modeling framework
NK: Natural killer
NMR: Nuclear magnetic resonance
PBMCs: Peripheral blood mononuclear cells
PCT: Procalcitonin;
*PF4*: Platelet factor 4
PLT: Platelet count
RRA: Robust rank aggregation
*S. aureus*: *Staphylococcus aureus*
SCENIC: Single-cell regulatory network inference and clustering
scRNA: Single-cell RNA
seqFISH-plus: Sequential fluorescence in situ hybridization-plus
SOFA: Sequential organ failure assessment
ST: Spatial transcriptomics
STRIDE: Spatial transcriptomics deconvolution by topic modeling
*S. viridans*: *Streptococcus viridans*
WBC: White blood cell count
WT: Wild type
