# Supplementary material for: Deciphering the molecular and cellular atlas of immune cells in septic patients with different bacterial infections
Source: J Transl Med. 2023 Nov 2;21:777. doi: 10.1186/s12967-023-04631-4 (PMC10621118; doi:10.1186/s12967-023-04631-4)
Supplement: Supplementary file 2 — Additional file 2: Table S2. The unique signature genes in septic patients with different bacterial infections. Figure S1. Dysregulated functions of other types. A Protein–protein interaction networks among the 143 genes. B and C The potential key gene sets identified from two datasets B and one dataset C. D Heatmap shows the function terms that were enriched in different types (Top 20 terms). Figure S2. The signature genes of other types. A Boxplots show the average expression of signature gene sets. P values are from a Wilcoxon test. B and C Receiver operating curves for out-of-sample prediction of case–control state B and differentiation between B. pseudomallei sepsis and others C trained on signature gene sets. Figure S3. The related cell clusters of other types. A The UMAP plot shows the origin of cells before (left) and after batch-correction (right) (harmony). B The density scatter plot shows the expression levels of all the signatures. The color gradient represents the enrichment score, with yellow indicating a higher score. Figure S4. The signature genes for other types of sepsis in related cell clusters. A The bar chart shows the relative cell abundance of C2_C1QA, C4_RPL34, C9_STOM and C13_ZNF703 in controls and septic patients. B Volcano plots depict the DEGs between C2_C1QA and C10_ULK1. c Functional enrichment of the DEGs between C2_C1QA and C10_ULK1. D-G Violin plots show the expression of signature genes in different clusters between sepsis and controls. H The dot plot shows the expression of signature genes of Micrococcus spp. sepsis in each cluster between sepsis and controls. P value is from a Wilcoxon test. Figure S5. Function of signature genes in related cell clusters. A qRT-PCR analysis of LDHA expression in a cohort enrolled in this study (controls, n = 4; sepsis patients, n = 5). P value is determined by unpaired Student's t-test. B The 2D image displays the RNA density (top) and the distributions of DRAM1 and its co-expressed genes (FAM49B, [file 12967_2023_4631_MOESM2_ESM.docx]

Additional file for

**Deciphering the molecular and cellular atlas of immune cells in septic patients with different bacterial infections**

Ping Sun^1, 2, a^, Mintian Cui^1, a^, Jiongjie Jing^1, a^, Fanyu Kong^3^, Shixi Wang^1^, Lunxian Tang^3^, Junling Leng^2^, Kun Chen^1,4*^

Correspondence to: chenk@tongji.edu.cn (K. C.).

These authors contributed equally: Ping Sun, Mintian Cui, and Jiongjie Jing.

**This file includes:**

Additional file Table 2

Additional file Figures 1 to 8.

**Additional file Table 2 The unique signature genes in septic patients with different bacterial infections**

| Pathogens | Genes |
| --- | --- |
| *A. baumannii* | *HIST1H4H*, *HNRNPA3P1*, *HPGD*, *IFI27*, *OSM*, *RAB20* |
| *B. pseudomallei* | *AC112496.1*, *AC139495.1*, *AL512785.2*, *AL596214.1*, *AL713999.1*, *ANKRD34B*, *AP5B1*, *ART3*, *BATF2*, *CARD17*, *CD274*, *COL17A1*, *DDAH2*, *DDIAS*, *ETV7*, *GBP6, GCH1, GPR42*, *LINC02555, MIR503HG*, *MYOF*, *PPM1N*, *SEPTIN4-AS1*, *SOCS1*, *TIFA* |
| *Corynebacterium spp.* | *FAM20C, LIPN, PPDPF, SLITRK4* |
| *E. coli* | *ACSL1*, *APMAP*, *BCL6*, *CD55*, *DRAM1*, *HDAC4*, *HIST1H1C*, *ITGAM*, *KLHL2*, *MTF1*, *NSUN7*, *PFKFB2*, *PLBD1*, *SERPINA1*, *SERPINB1*, *SH3GLB1*, *SLC22A15*, *SULT1B1*, *VPS9D1* |
| *Group A strep* | *ACER3*, *ATP9A*, *ERLIN1*, *FBXO6*, *GLT1D1*, *HIST1H2BD*, *SIGLEC9*, *TOP2A* |
| *Group B strep* | *AGPAT9*, *CKAP4*, *CPEB4*, *DAAM2*, *FGF13*, *LOC101928429*, *MS4A3, OPLAH*, *PADI4*, *PRTN3*, *REG1B*, *SERPINB10*, *TSPO, TXN* |
| *K. pneumoniae* | *CADM3-AS1*, *DEFA1*, *PTPN20* |
| *Micrococcus spp.* | *AC012447.1*, *ANK1*, *ASPRV1*, *BAMBI*, *C1orf198*, *EPHB4*, *ESAM*, *GYPA*, *GYPE*, *HACD1*, *HBD*, *HBG2*, *HSPB1P1*, *HTRA1*, *LONRF3*, *LSMEM1*, *MAOA*, *MAP1A, MARC1*, *MMRN1*, *PF4V1*, *SAMD14*, *SELP*, *TGFB1I1*, *TMEM176A* |
| *Neissera* | *C15orf48*, *C1QA*, *IL10RB-AS1*, *IL1RN*, *PI3* |
| *Salmonella* | AC092757.2, ASGR2, BCAT1, CDC42EP3, CDC45, *CDCA5*, *CLEC4E*, *CXCR6*, *GASK1B*, *GPER1*, *IL1R1-AS1, LILRB4*, *MARCO*, *MILR1, MT1E*, *NETO2*, *PCLAF*, *PPARG*, *RGL1*, *SLC2A11*, *SLC39A8*, *STAB1*, *TMIGD3* |
| *S. viridans* | *B4GALT5*, *BEX1*, *CTSD*, *CTSG*, *DNAJC3*, *FNDC3B*, *PTX3*, *RNASE3*, *SUCNR1* |

**Additional file Figures**

^
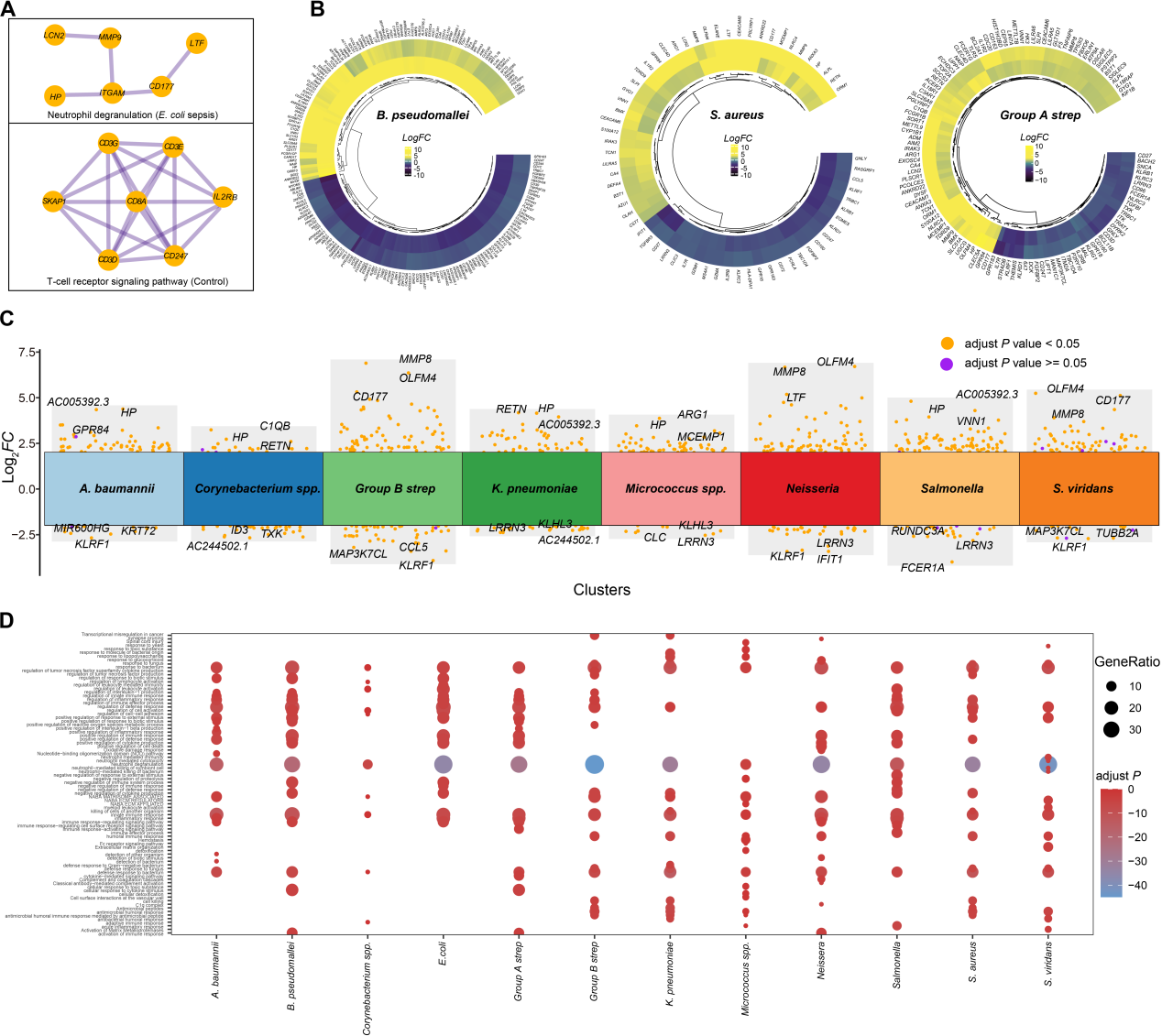
^

**Additional file Fig. 1 Dysregulated functions of other types. A** Protein-protein interaction networks among the 143 genes. **B and C** The potential key gene sets identified from two datasets **B** and one dataset **C. D** Heatmap shows the function terms that were enriched in different types (Top 20 terms).


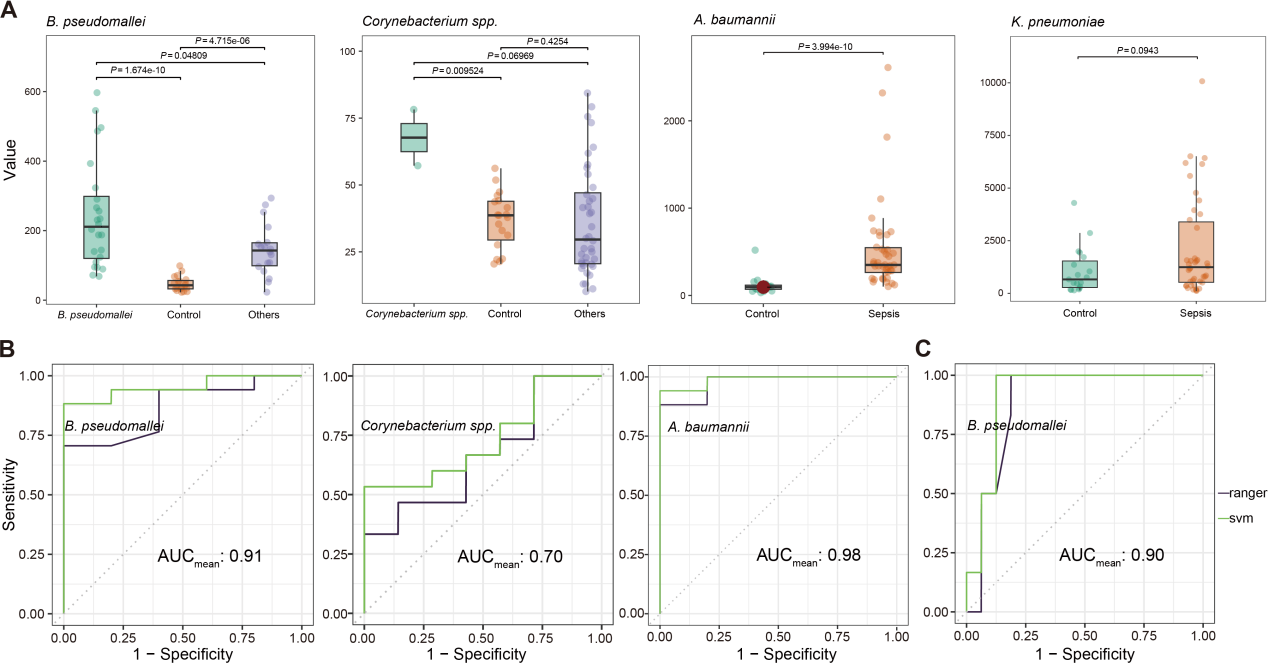


**Additional file Fig. 2 The signature genes of other types. A** Boxplots show the average expression of signature gene sets. *P* values are from a Wilcoxon test. **B and C** Receiver operating curves for out-of-sample prediction of case-control state **B** and differentiation between *B. pseudomallei* sepsis and others **C** trained on signature gene sets.


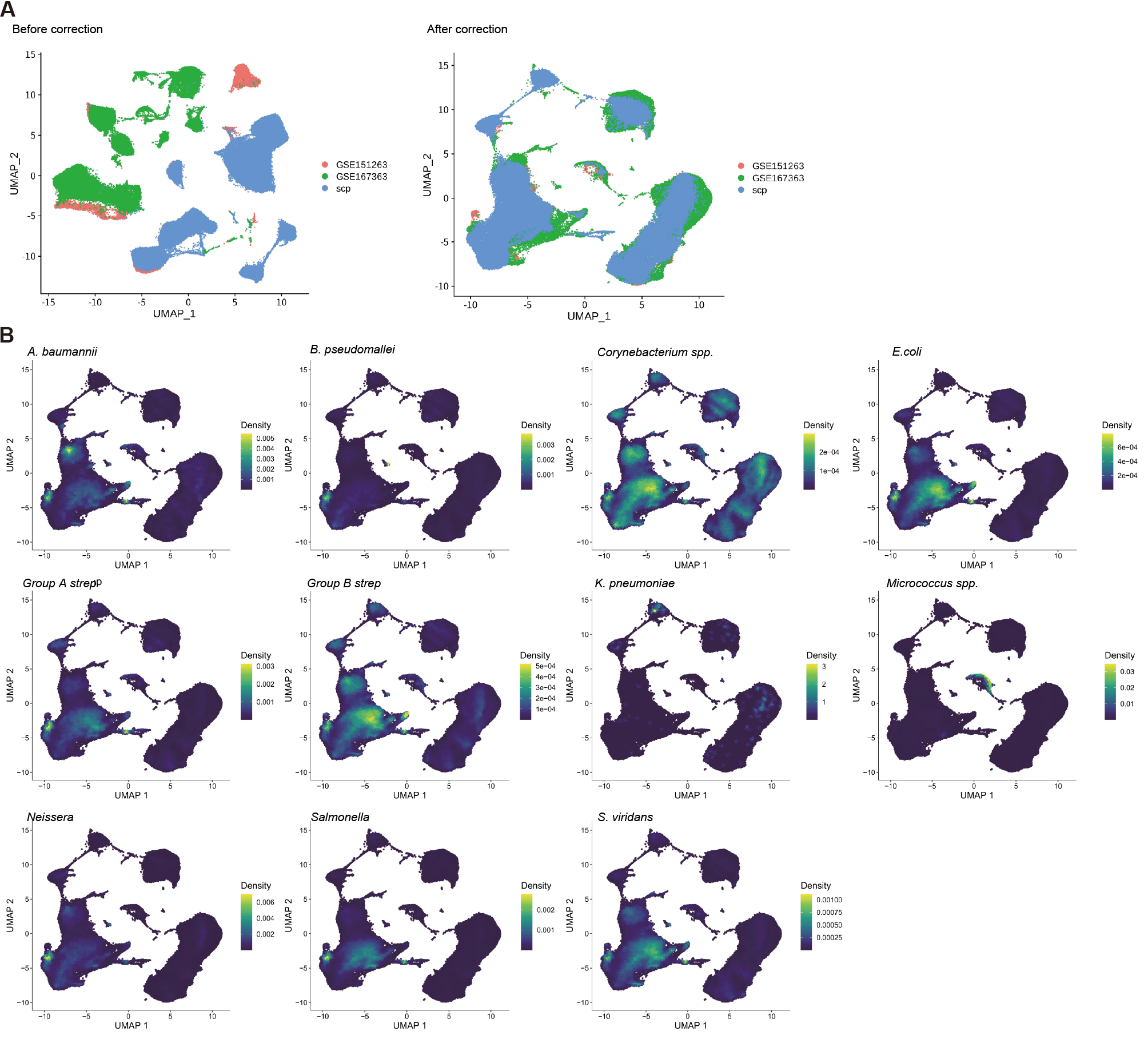


**Additional file Fig. 3 The related cell clusters of other types. A** The UMAP plot shows the origin of cells before (left) and after batch-correction (right) (harmony). **B** The density scatter plot shows the expression levels of all the signatures. The color gradient represents the enrichment score, with yellow indicating a higher score.

^
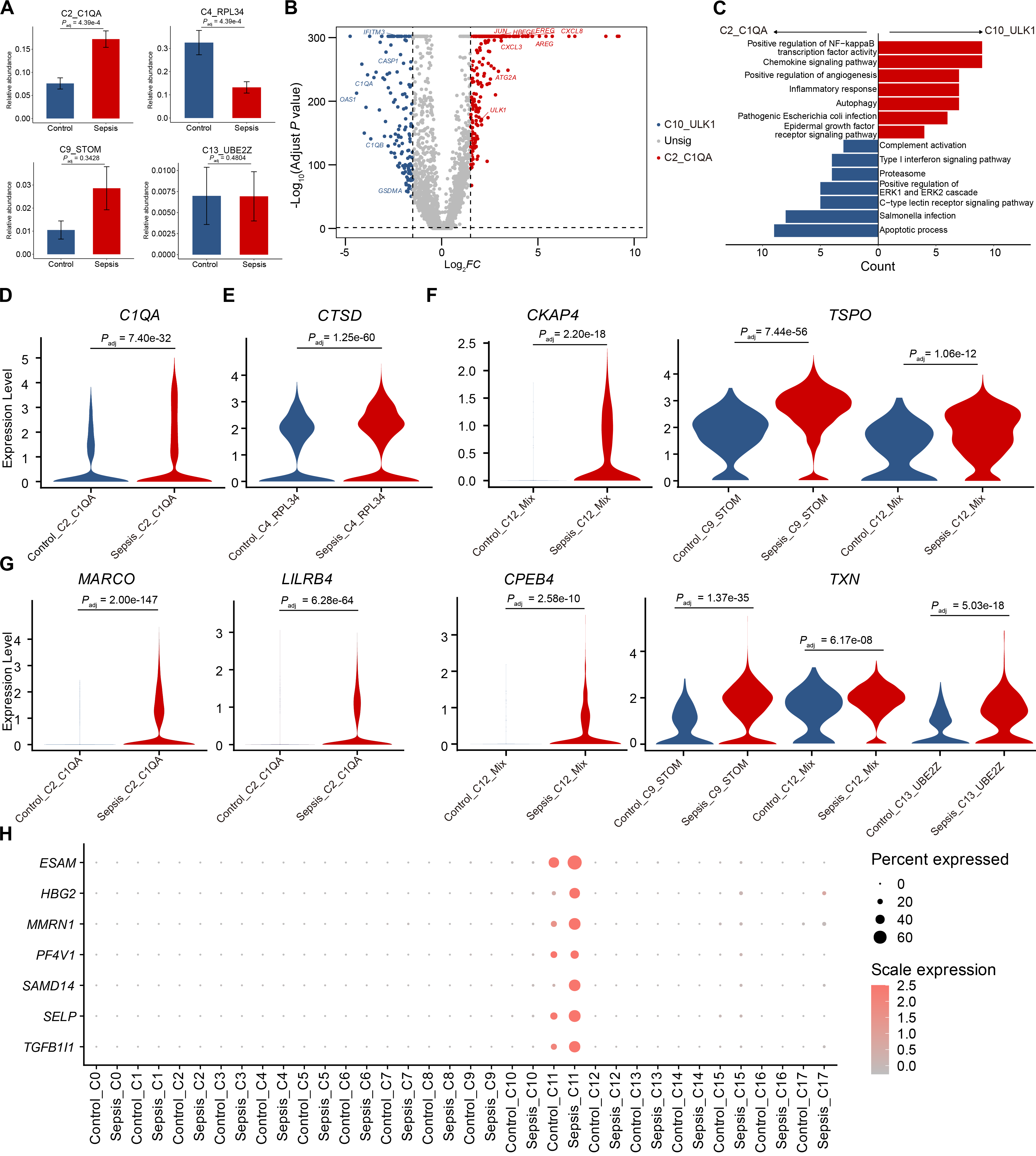
^

**Additional file Fig. 4 The signature genes for other types of sepsis in related cell clusters. A** The bar chart shows the relative cell abundance of C2_C1QA, C4_RPL34, C9_STOM and C13_ZNF703 in controls and septic patients. **B** Volcano plots depict the DEGs between C2_C1QA and C10_ULK1. **c** Functional enrichment of the DEGs between C2_C1QA and C10_ULK1. **D-G** Violin plots show the expression of signature genes in different clusters between sepsis and controls. **H** The dot plot shows the expression of signature genes of *Micrococcus spp.* sepsis in each cluster between sepsis and controls. *P* value is from a Wilcoxon test.

^
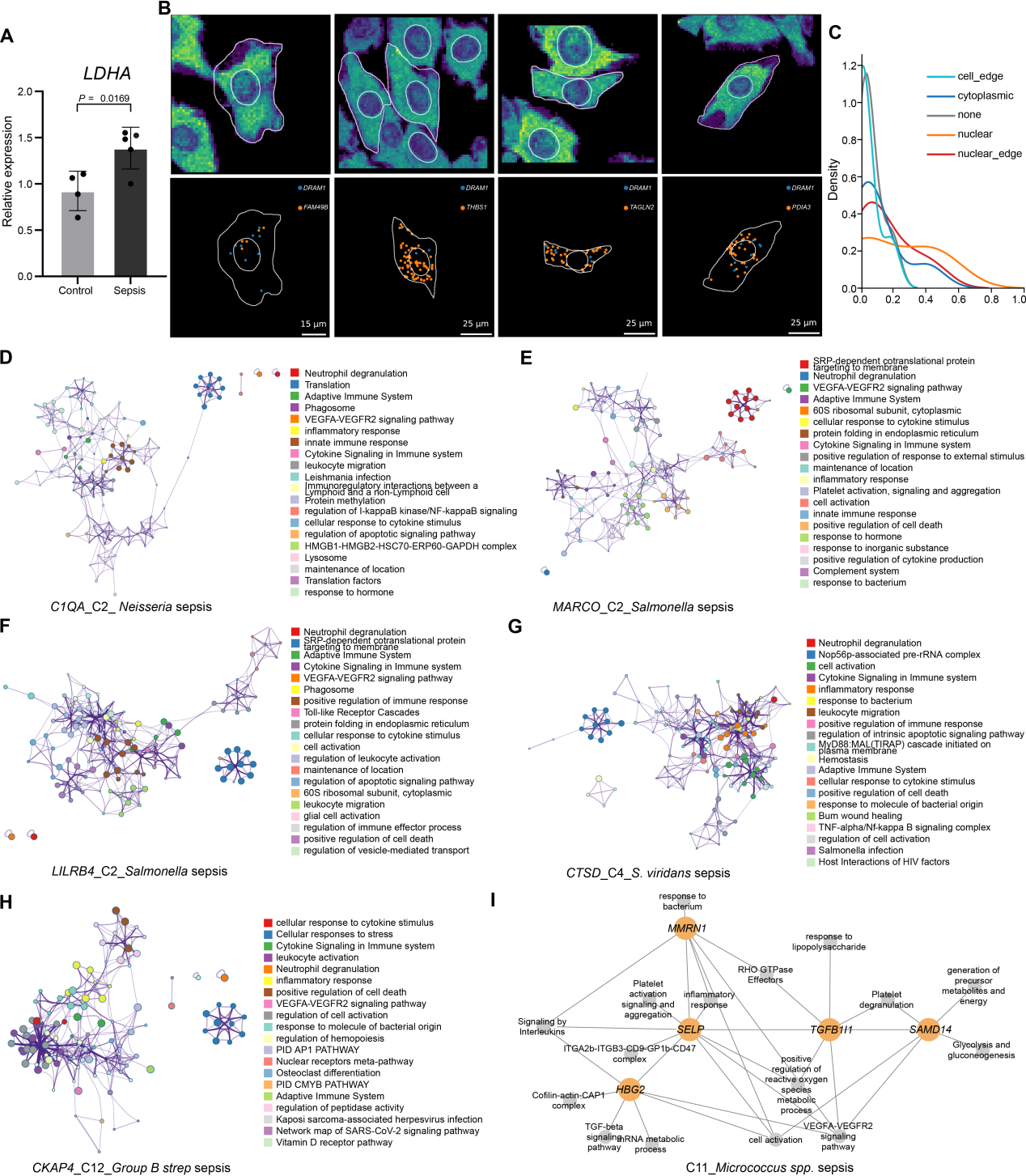
^

**Additional file Fig. 5 Function of signature genes in related cell clusters. A** qRT-PCR analysis of LDHA expression in a cohort enrolled in this study (controls, n = 4; sepsis patients, n = 5). *P* value is determined by unpaired Student's t-test. **B** The 2D image displays the RNA density (top) and the distributions of *DRAM1* and its co-expressed genes (*FAM49B*, *THBS1*, *TAGLN2*, and *PDIA3*) (bottom). **C** The density distribution plot illustrates the regions where *DRAM1* and its adjacent five genes are distributed within the cells**. D-H** Network of enriched terms by co-expressed genes of signature genes in related clusters. **I** *HBG2*, *MMRN1*, *SAMD14*, *SELP*, and *TGFB1I1* are involved in sepsis-related functions in C11_plate.

^
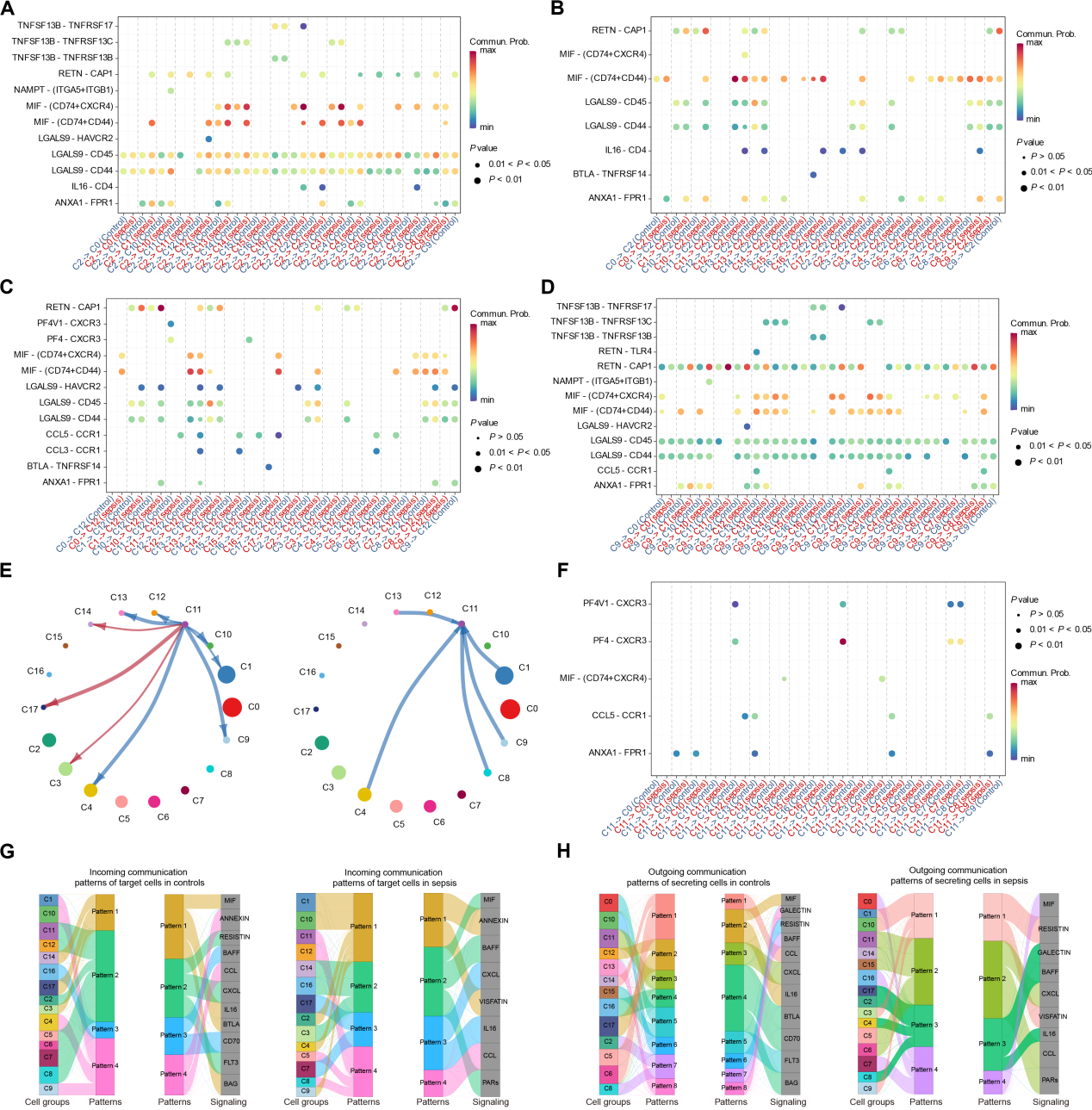
^

**Additional file Fig. 6 Cell-cell communication among cell clusters in PBMCs. A-D and F** Comparison of significant ligand-receptor pairs was conducted between C2_C1QA (outgoing) and other clusters (incoming) **A**, between other clusters (outgoing) and C2_C1QA (incoming) **B**, between other clusters (outgoing) and C12_mix (incoming) **C**, between C9_STOM (outgoing) and other clusters (incoming) **D**, and between C11_plate (outgoing) and other clusters (incoming) **F**, in both controls and septic patients. **e** Differential interaction strength between C11_plate and other cell clusters. **G and H** Inferred incoming and outgoing communication patterns of secreting cells between controls and septic patients. The inferred potential patterns are correspondent to cell populations and signaling pathways. The thickness of the flow indicates the contribution of the cell group or signaling pathway to each potential pattern.

**
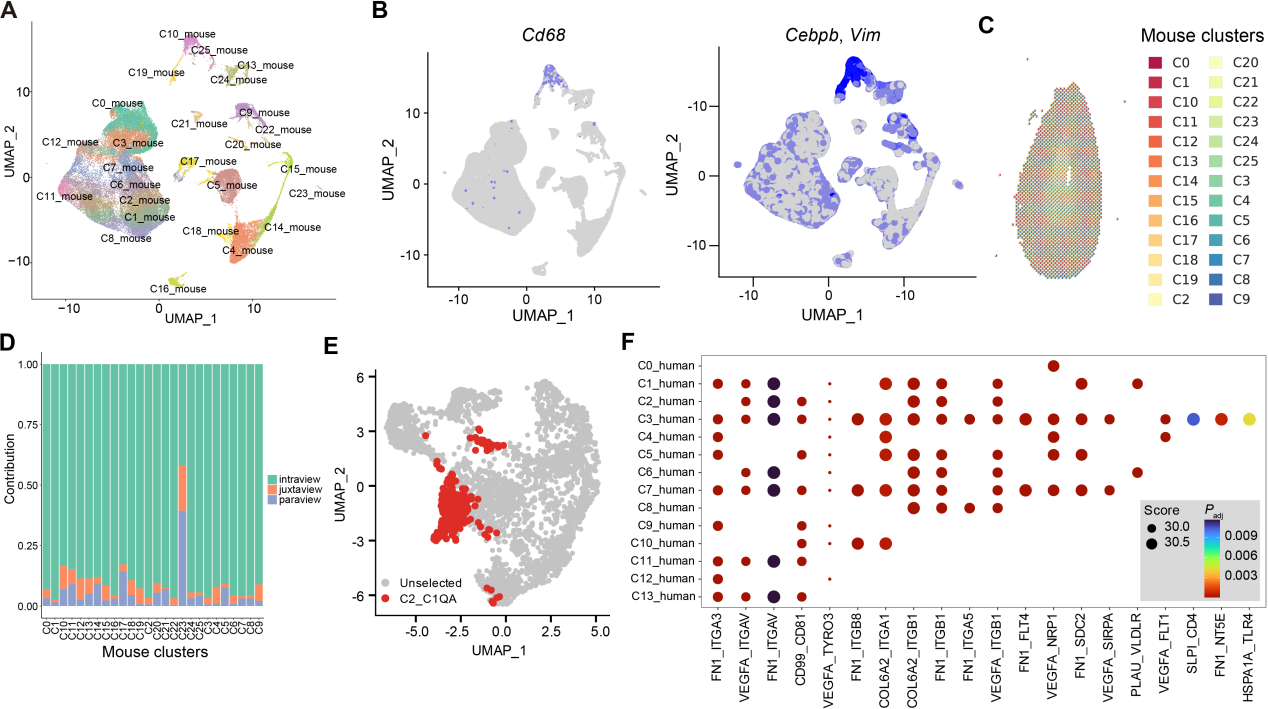
Additional file Fig. 7 Cell-cell communication among cell clusters in kidney tissue. A** The UMAP plot shows scRNA data from kidney tissue of control mice and sepsis model mice. **B** The UMAP plots show the marker genes of macrophage and C10_ULK1 in mouse kidney tissue. **C** The scatter pie plot shows the spatial locations of different cell types predicted by STRIDE. Colors represent different cell types. **D** The relative contribution of each view to the prediction of cell interactions. **E** The UMAP plot displays spots that are predicted to be C2_C1QA in the spatial data of human kidney tissue. **F** The identified ligand-receptor pairs between C2_human and other clusters.

**
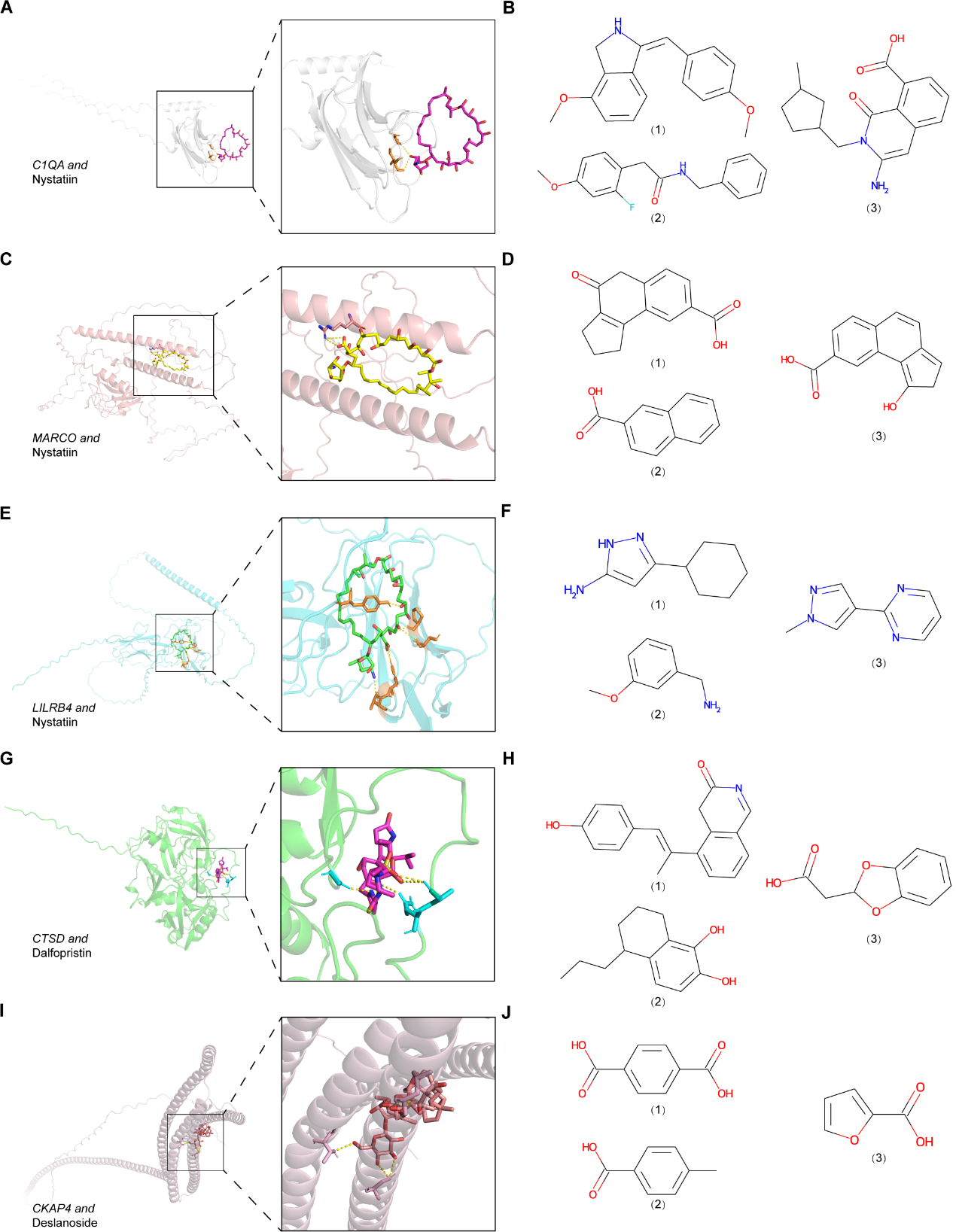
**

**Additional file Figure 8 Identification of potential target drugs and molecules. (A, C, E, G, and I)** (Left) Protein-ligand docking complex. (Right) Zoomed-in views of the interaction contact region. **(B, D, F, H, and J)** *De novo* molecular design based on C1QA **(B)**, MARCO **(D)**, LILRB4 **(F)**, CTSD **(H)**, or CKAP4 **(J)**.
